# New Hope for Treating Intervertebral Disc Degeneration: Microsphere-Based Delivery System

**DOI:** 10.3389/fbioe.2022.933901

**Published:** 2022-07-19

**Authors:** Taowen Guo, Xiaobo Zhang, Yicun Hu, Maoqiang Lin, Ruihao Zhang, Xiangyi Chen, Dechen Yu, Xin Yao, Peng Wang, Haiyu Zhou

**Affiliations:** ^1^ Department of Orthopedics, Lanzhou University Second Hospital, Lanzhou, China; ^2^ Key Laboratory of Bone and Joint Disease Research of Gansu Province, Lanzhou, China; ^3^ Department of Spine Surgery, Honghui Hospital, Xi’an Jiaotong University, Xi’an, China; ^4^ Xigu District People’s Hospital, Lanzhou, China

**Keywords:** intervertebral disc degeneration, mechanism, microspheres, delivery system, inflammation, regeneration, treatment

## Abstract

Intervertebral disc (IVD) degeneration (IVDD) has been considered the dominant factor in low back pain (LBP), and its etiological mechanisms are complex and not yet fully elucidated. To date, the treatment of IVDD has mainly focused on relieving clinical symptoms and cannot fundamentally solve the problem. Recently, a novel microsphere-based therapeutic strategy has held promise for IVD regeneration and has yielded encouraging results with *in vitro* experiments and animal models. With excellent injectability, biocompatibility, and biodegradability, this microsphere carrier allows for targeted delivery and controlled release of drugs, gene regulatory sequences, and other bioactive substances and supports cell implantation and directed differentiation, aiming to improve the disease state of IVD at the source. This review discusses the possible mechanisms of IVDD and the limitations of current therapies, focusing on the application of microsphere delivery systems in IVDD, including targeted delivery of active substances and drugs, cellular therapy, and gene therapy, and attempts to provide a new understanding for the treatment of IVDD.

## 1 Introduction

Low back pain (LBP) has become the most common public health problem worldwide in recent years and can occur in people of almost all ages (Hartvigsen et al., 2018). It not only seriously affects daily life but also places a heavy burden on social health care and even economic development. Relevant authoritative survey reports show that the global prevalence of LBP (0–100 years old) is 9.4%, and it is the main disease that causes disability, overall economic burden, and demand for rehabilitation services worldwide (Hoy et al., 2014; Hartvigsen et al., 2018; Cieza et al., 2021). With the growth of the global population and the acceleration of population aging, the disability and treatment costs caused by LBP will increase in the future. Although the pathogenic factors of LBP are complex and diverse, intervertebral disc (IVD) degeneration (IVDD) is considered to be the most significant (Zhang W. et al., 2022). IVDD is also a result of multivariate integrated effects, such as aging, genetics, abnormal mechanical load, circadian rhythm disorder, obesity, and smoking (Cazzanelli and Wuertz-Kozak, 2020; Cheng et al., 2021; Morris et al., 2021; Zhang et al., 2021). Currently, IVDD can be treated by nonsurgical and surgical methods according to the severity of the disease, but generally, these can only temporarily alleviate clinical symptoms and cannot prevent the progression of degeneration. Given the increasing number of patients with IVDD and the absence of more satisfactory treatment measures, the development of new treatment options has become a pressing challenge.

In recent years, with the rapid development of tissue engineering technology and the continuous discovery of new delivery materials, tissue engineering strategies for IVD repair and regeneration have attracted much attention. Microspheres, as flowable spherical particles capable of loading specific substances with good biocompatibility and biodegradability, can be injected into the IVD and slowly release the loaded substances locally to exert a sustained therapeutic effect (Kulchar et al., 2021). This microsphere-based delivery system alleviates LBP, inhibits tissue degeneration, and promotes IVD regeneration or repair through the targeted delivery of cells, drugs, bioactive components, and gene modifiers to the interior of IVD tissue (Feng et al., 2017; Xia et al., 2019; Xu H. et al., 2020; Wiersema et al., 2021). This novel microsphere therapy breaks the traditional treatment situation and may further restore the IVD anatomical structure and biological function based on the inhibition of IVDD progression, showing great potential for development in the field of IVD tissue engineering. This article first reviews the physiological structure and degeneration mechanism of IVD and summarizes the current treatment strategies for IVDD and their drawbacks. Then, we introduced the application of microsphere delivery systems in IVDD in detail and analyzed the current research status and some shortcomings. We hope that this study can help researchers systematically understand the application of microsphere delivery systems in IVDD and provide a theoretical basis for exploring more advanced microsphere delivery systems for IVD regeneration.

## 2 Mechanisms and Treatment Strategies of IVDD

### 2.1 Structure and Function of IVD

**FIGURE 1:**
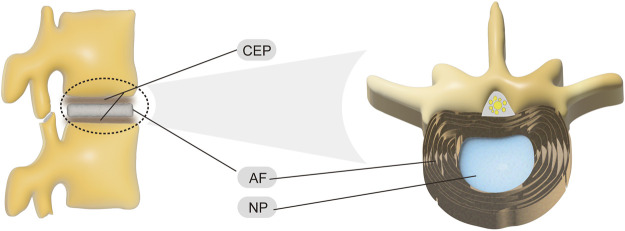
Structure of normal IVD.

The IVD is the largest avascular structure in the human body (Sun et al., 2020), and its structures include the centrally located nucleus pulposus (NP), the external annulus fibrosus (AF), and the cartilaginous endplates (CEPs) at the upper and lower ends (Figure 1). Among them, NP is a highly hydrated gel with a water content of more than 80% and consists mainly of notochord cells, NP cells (NPCs), type II collagen (COL2), and proteoglycans (PG) (Li et al., 2021). Due to this characteristic, the NP tissue can evenly distribute the force to the AF when the IVD is pressed, providing excellent cushioning performance (Heo and Park, 2022). AF is a fibrous structure wrapped around the NP that is composed of 15–25 concentric annular lamellae containing abundant elastin fibers, fibroblasts, and chondrocytes (Molladavoodi et al., 2020). Interestingly, the composition of the AF-NP transition zone is close to that of NP, forming a buffer zone with soft traits (Guerin and Elliott, 2007). These functional structures of the AF protect and fix the internal NP tissue while possessing good elasticity, which is essential for the reset of the IVD shape. The CEP is the demarcation line between the IVD and the vertebrae, with an average thickness of approximately 1 mm, and it shows a thick peripheral and thin intermediate layer of hyaline cartilage (Wu et al., 2021). It is worth noting that the micropores in the center of the CEP have a permeability function, which facilitates the exchange of substances between the vertebrae and the IVD and has the function of preventing the loss of NP liquid and maintaining the fluid pressure, maintaining the balance between the mechanical properties and the nutritional requirements of the IVD (Zehra et al., 2015).

In short, the IVD is a sandwich-like structure that, together with the joints between the vertebrae and the attached muscles and ligaments, forms a composite motion cushioning system that is subject to compression, tension, rotation, and bending (Li et al., 2021). The IVD acts as a ball bearing in this movement system, playing a vital role in increasing the range of motion of the spine, buffering pressure and vibration, and protecting the brain and spinal cord.

### 2.2 Pathogenesis of IVDD

**FIGURE 2:**
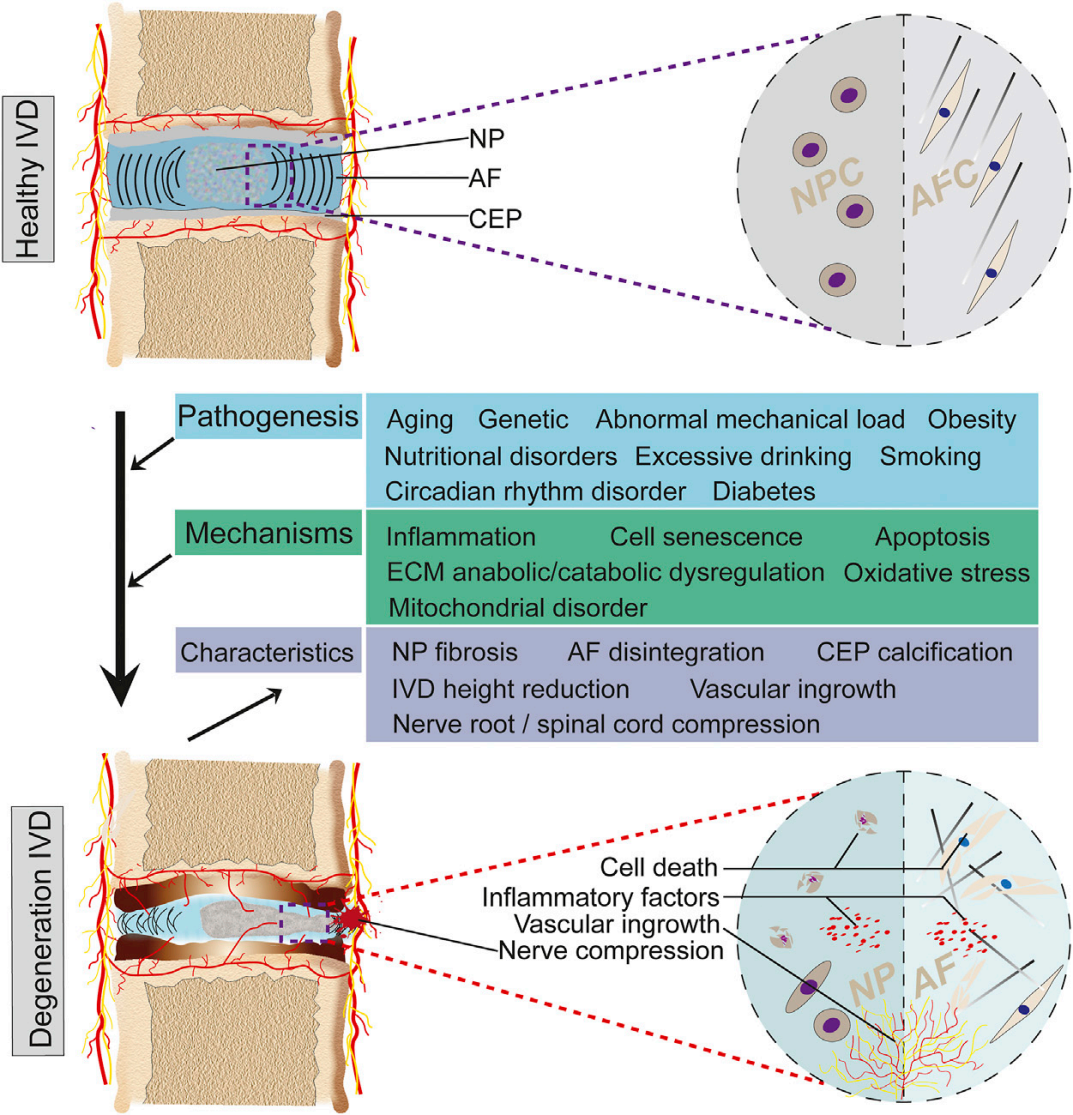
Degenerative process of IVD. IVD gradually degenerates in response to multiple pathogenic factors. Pathogenic factors include aging, genetics, nonphysiological mechanical loading, nutritional disorders, etc. These factors ultimately lead to IVDD through pathological mechanisms such as inflammation, apoptosis, ECM anabolic/catabolic dysregulation and oxidative stress. The degenerative process exhibits a series of features such as highly decreased IVD, NP fibrosis, AF disintegration, and vascular ingrowth.

The development of IVDD is exceptionally complex and, in general, is the result of a combination of age, genetics, nonphysiological mechanical load, nutritional disorders, and circadian rhythm disturbances (Wang et al., 2016; Patil et al., 2019; Cazzanelli and Wuertz-Kozak, 2020; Cheng et al., 2021; Morris et al., 2021). The above factors cause a series of pathological changes through apoptosis, inflammation, extracellular matrix (ECM) degradation, oxidative stress, and mitochondrial dysfunction, including reduction of IVD cells, loss of cellular physiological functions, and disturbance of ECM metabolism, which in turn lead to NP fibrosis, AF disintegration, and CEP calcification, ultimately resulting in loss of IVD mechanical properties, IVD height reduction, and NP protrusion (Feng et al., 2016; Kang et al., 2019; Lin et al., 2020; Xu G. et al., 2020; Zhang Y. et al., 2020). Clinical symptoms such as LBP and lower limb numbness will occur when the spinal cord and spinal nerve roots are compressed (Figure 2).

As age increases, the high water-holding notochord cells are gradually replaced by other IVD cells, and a decrease in the water content of NP tissue is an early manifestation of IVDD (McCann et al., 2012). In addition, aging is accompanied by an increase in the senescence-associated secretory phenotype (SASP), which inhibits ECM synthesis and promotes the expression of multiple matrix metalloproteinases (MMPs) through inflammatory factors such as interleukin (IL)-1β, IL-6, IL-8, and tumor necrosis factor (TNF)-α, ultimately disrupting the IVD microenvironment and homeostasis (Feng et al., 2016; Patil et al., 2019; Birch and Gil, 2020). Furthermore, mitochondrial dysfunction and oxidative stress are common to organismal aging. Oxidative stress can induce NPC apoptosis by promoting excessive mitochondrial autophagy, which leads to IVDD (Xu W.-N. et al., 2019). In addition to aging, the relationship between genetic factors and IVDD has also been studied by researchers. The classic twin experiment confirmed the genetic correlation of IVDD and LBP to some extent (Battié et al., 1995; Battié et al., 2007). Moreover, genetic polymorphism studies may also provide a basis for this, as genetic polymorphisms in inflammatory factors (IL-1α/β, IL-6, and TNF-α), MMPs (MMP-1, -2, -3), collagen (COL), aggrecan (ACAN), and cartilage intermediate proteins may be associated with the pathogenesis of IVDD in populations from several countries (Mayer et al., 2013; Harshitha et al., 2018; Trefilova et al., 2021). It is well known that both prolonged compression, strain, and acute nonphysiological loading acting on the IVD can lead to a range of changes, such as tissue damage, reduced vascular buds, decreased cellular activity, inflammatory factor release, and ECM degradation (Alkhatib et al., 2014; Zhan et al., 2020; Zhan et al., 2021; Zhou et al., 2021), which ultimately result in IVDD. Disregarding exogenous factors, the IVD is a hypoxic, low nutrient acidic microenvironment due to the absence of blood supply (Bibby et al., 2005). This tissue ecological niche may not be conducive to cellular metabolic activity. For example, AF cells (AFCs) exhibit suppressed proliferation and metabolic activity as well as apoptosis and senescence in the presence of nutritional deficiency (Yurube et al., 2019). Moreover, a chronic low nutritional state can further accelerate IVDD progression (Gu et al., 2014). Therefore, nutritional availability should be taken into account in the treatment of IVDD. In addition, circadian rhythm disturbance also induces IVDD, which may be related to excessive autophagy within the IVD (Zhang T.-W. et al., 2020; Morris et al., 2021).

### 2.3 Nonsurgical Strategies

Patients with IVDD usually resort to exercise therapy, physical means, drug therapy, and block therapy for pain relief in the early and middle stages of the disease. First, exercise therapy requires patients to strengthen their back muscles with physical exercise, including tai chi, yoga, Pilates, and whole-body vibration exercises, which can improve symptoms and reduce disability rates (Luan et al., 2019; Wu et al., 2020). Animal studies suggest that physical activity may reduce pain by repairing IVD and increasing cell density and that high-load exercise patterns at low volume and frequency may promote IVD regeneration (Luan et al., 2015; Steele et al., 2015). However, while exercise can be therapeutic, it should also be done scientifically. Second, some physical means, including local hot compress, massage, Tui-Na, traction, and physiotherapy (shock wave, laser, ultrasound, etc.), also have better relief during acute attacks of LBP (Zhao et al., 2019). They are commonly used for pain relief and rehabilitation. Third, pharmacological treatment is essential for the management of chronic LBP. Currently, nonsteroidal anti-inflammatory drugs (NSAIDs), analgesics (e.g., paracetamol and opioids), omega-3 fatty acids, and muscle relaxants play an important role in the treatment of pain of IVD origin (NaPier et al., 2019; Gkantsinikoudis et al., 2022). However, NSAIDs can cause severe gastrointestinal discomfort, and therefore a comprehensive evaluation of the efficacy and adverse effects of such drugs should be made before choosing them (McEvoy et al., 2021). Finally, when the patient has a more limited pain point, closed treatment measures such as epidural injection of corticosteroids may be used, but this is short-lived (Kanayama et al., 2015). It is worth mentioning that acupuncture, a traditional Chinese medicine treatment, is considered to be better at relieving pain, but some argue that the evidence is insufficient (Standaert et al., 2011). In brief, nonsurgical strategies demonstrated better results in the relief and rehabilitation of LBP but could not stop the degenerative process.

### 2.4 Surgical Strategies

**FIGURE 3:**
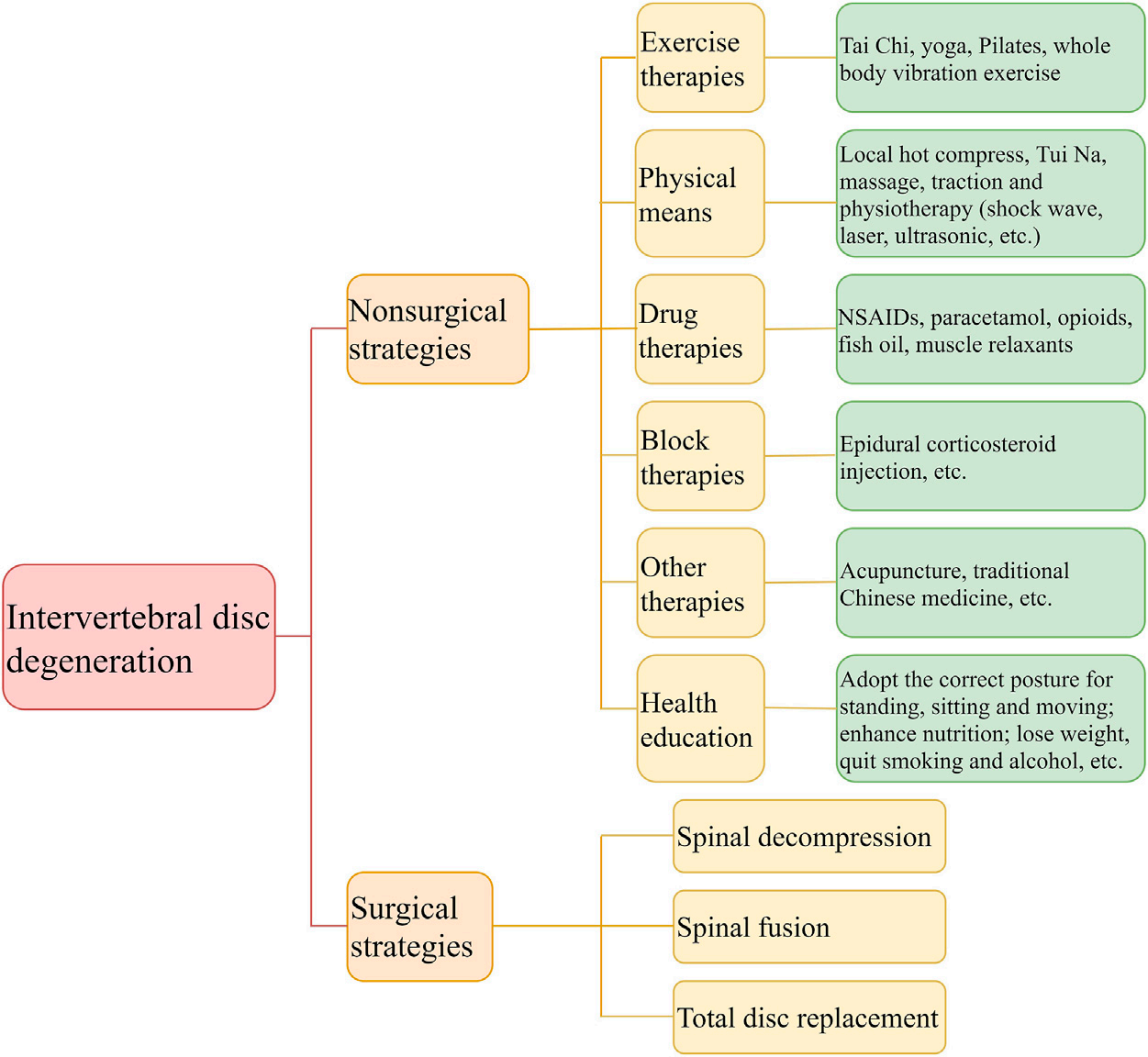
Nonsurgical and surgical strategies for IVDD.

IVDD patients often opt for surgical treatment when conservative treatment fails, including spinal decompression, spinal fusion, and total disc replacement (TDR). With the improvement of surgical techniques and the advancement of instruments, lumbar decompression has been minimally invasive, which has greatly shortened the postoperative recovery time of patients (Aihara et al., 2022). The number of lumbar fusions chosen for IVDD, IVD herniation, and spinal stenosis in the United States accounted for 42.3% of the total number of this procedure in 2015 (Martin et al., 2019). Overall, spinal fusion has become the first choice for patients with IVDD, but complications such as slow postoperative recovery, infection, LBP recurrence, fusion failure, cage subsidence, and adjacent segmental disease also pose significant problems for patients and clinicians (Eliasberg et al., 2016; Moser et al., 2022; Zhang N.-Z. et al., 2022). TDR has generated a great deal of interest in spine surgery as an innovative surgical procedure that allows the use of artificial IVDs to replace IVDs that have degenerated or lost function in the human body. Although this IVD prosthesis, prepared from metal and polymeric materials, has good biological inertness, the fragments formed by long-term wear can trigger an immune-inflammatory response in the surrounding tissues, leading to bone loss around the implant, which can result in the loosening of the prosthesis or even surgical failure in the long run (Lin et al., 2014; Werner et al., 2018). Furthermore, persistent pain in periprosthetic tissues caused by inflammatory responses and biomechanical loading changes, as well as complications such as infection, facet arthrosis, and heterotopic ossification, poses a challenge to the promotion of TDR (Beatty, 2018; Park et al., 2018; Werner et al., 2018) (Figure 3). In conclusion, any invasive procedure will destroy the original structure and have corresponding postoperative complications that can have serious consequences if not handled properly.

### 2.5 Early Exploration and Dilemma of IVD Regeneration Strategies

As mentioned above, neither conservative nor surgical treatments can fundamentally stop or reverse the IVDD process. In search of a breakthrough, a large number of researchers have explored biomedical approaches for IVD tissue regeneration/repair, such as direct growth factor injection, cell transplantation, and gene therapy (Buser et al., 2019). Although some regenerative medicine therapies have been attempted in humans, most have struggled to achieve clinical translation because of poor outcomes, risks, and lack of long-term efficacy. While successful animal experiments have demonstrated that direct pushing of growth factors such as osteogenic protein 1 (OP-1) contributes to IVD height and structural recovery, the regenerative potential of OP-1 in an *in vitro* culture model of human degenerated NP tissue shows donor dependency and high dose requirements, which increases the probability of ectopic ossification (Masuda et al., 2006; van Dijk et al., 2017). Moreover, the anabolic capacity of IVD cells decreases during degeneration, and thus direct injection of growth factors may be effective only for mild IVDD (Paesold et al., 2007). Autologous cell transplantation may be a reliable treatment for IVDD, but the implantation of autologous IVD chondrocytes in animal and clinical models has only reaped some success in the short term (2 years), and its long-term efficacy has not been validated (Meisel et al., 2007; Hohaus et al., 2008). Additionally, the increased risk of unnecessary infection and injury associated with the surgical acquisition of autologous IVD cells and the fact that cells obtained from degenerated IVDs may not always function properly after implantation are issues that need to be addressed. Similarly, stem cell transplantation has been considered promising for curing IVDD. However, direct injection of mesenchymal stem cells (MSCs) within degenerative IVDs often induces osteophytes due to leakage (Vadalà et al., 2012). It is evident that there are disadvantages to direct injection and that a proper cell carrier is necessary. From inefficient naked DNA implantation to gene carrier transplantation, gene therapy is gradually being improved and used for innovative treatment of various diseases (Wolff et al., 1990; Paesold et al., 2007). The option of genetic modification of IVD cells to increase the supply of matrix components and beneficial substances has also been attempted to delay or even reverse IVDD. However, viral vectors may lead to insertional mutations and immune and inflammatory responses, and nonviral vectors mostly suffer from inadequate transfer and translation efficiency, limiting the progress of early gene therapy in clinical applications (Li et al., 2021). This shows that there are limitations of early IVD regeneration therapies, and it is necessary to improve upon them to explore truly effective IVD regeneration strategies.

## 3 Application of Microsphere-Based Delivery Systems in IVDD

**FIGURE 4:**
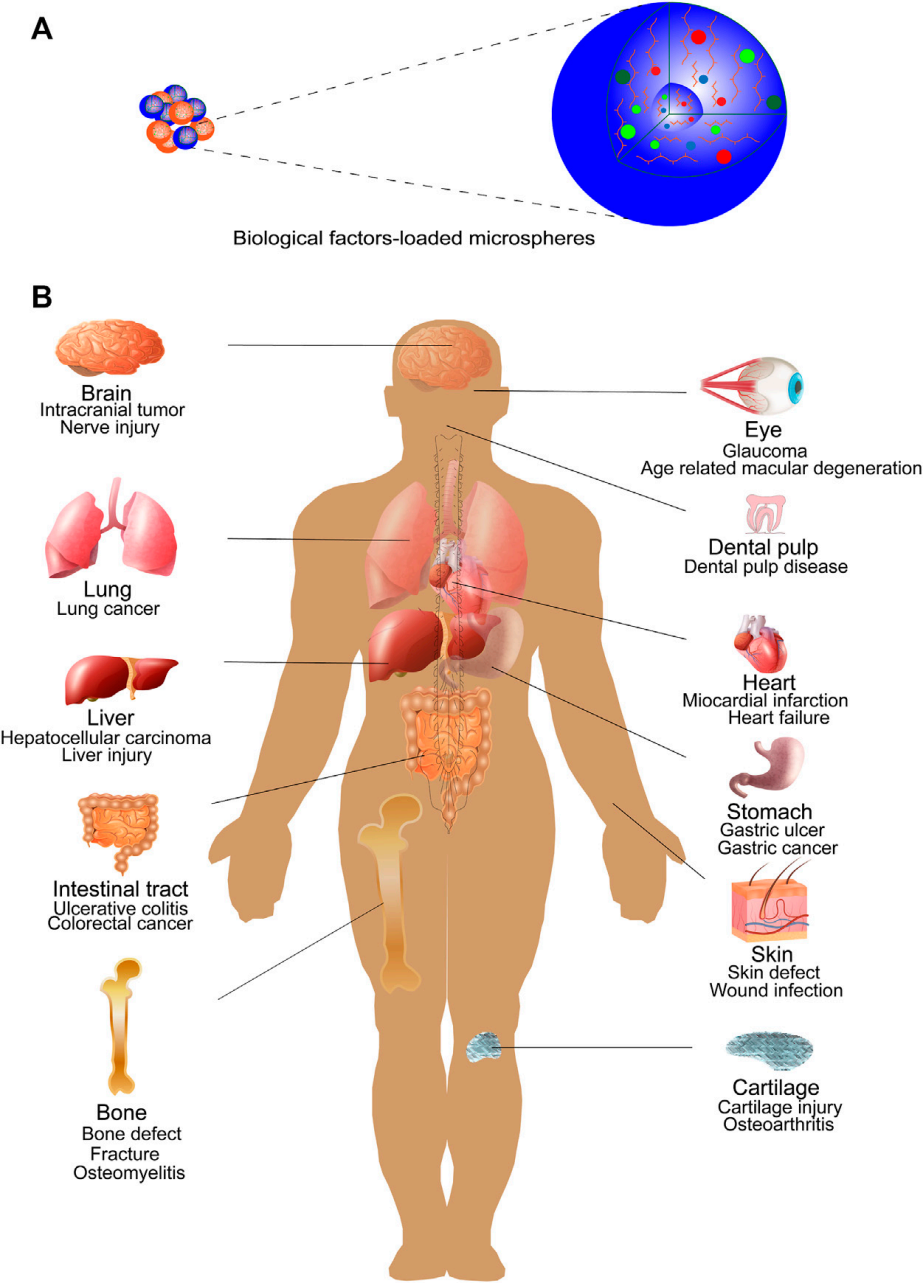
Biomedical applications of microspheres. **(A)** Microspheres loaded with various biological factors (e.g., growth factors, drugs). **(B)** Microspheres have been extensively studied and used in biomedical fields as delivery and slow release carriers of biologic factors. Studies mainly focus on the repair of various tissue or organ injuries and the treatment of some tumors. Example include bone, cartilage, brain tissue, liver, lung, heart, gastrointestinal tract, eye, skin, dental pulp, etc.

Microspheres are a class of three-dimensional (3D) spherical structures with an average particle size of 1–1,000 μm that are widely used in biomedical fields for their good substance delivery (Gupta et al., 2017) (Figure 4). Microspheres include both microcapsules and micromatrices, where microcapsules tend to have a “core-shell” structure consisting of a core of therapeutic substance and a shell of material, while micromatrices are formed when the therapeutic component is dispersed or embedded in the entire material matrix (Virmani and Gupta, 2017). Currently, the materials used to make microspheres are mainly organic polymers, such as natural polymers and synthetic polymers. Natural polymers, including gelatin (Tsaryk et al., 2015), collagen (Li et al., 2014), chitosan (Zhang H.-Y. et al., 2022), alginate (Hassani et al., 2022; Zhang H.-Y. et al., 2022), and starch (Ranjbar et al., 2022), possess excellent biocompatibility, biodegradability, and low toxicity. Among the synthetic polymers, poly(lactic-co-glycolic acid) (PLGA) (Shi et al., 2022), poly(l-lactic acid) (PLLA) (Xu Y. et al., 2020), and poly(ester amide) (PEA) (Tellegen et al., 2018) have good degradability, modifiability, and mechanical stability.

Thus far, novel microsphere delivery systems formed by loading therapeutic substances onto polymeric materials have shown great therapeutic potential. For example, this delivery system may reduce dose and toxicities and improve safety, efficacy, and patient compliance (Agnihotri et al., 2019). Therapeutic substances used for IVDD include drugs, active substances (mainly proteins or peptides), and gene regulatory factors (Feng et al., 2017; Xu H. et al., 2020; Wiersema et al., 2021). Among them, microcapsules/micromatrices of drugs, peptides, and proteins can be fabricated by various methods, such as emulsion-solvent evaporation, spray drying, phase separation, electrospraying, and microfluidic technology, each with its own advantages and disadvantages (Gupta et al., 2017; Ghosh Dastidar et al., 2018; Yang et al., 2019) (Table 1). Importantly, the water-oil-water (W/O/W) multiple emulsification method is the most popular because it can better protect the activity of substances in the internal aqueous phase. Encapsulation of peptides and proteins in microspheres is extremely challenging because their biological activity depends on the complex and ordered spatial structure, so their biological activity must be strictly maintained both in the acquisition of peptides/proteins and in the fabrication of microspheres. On the other hand, cells for therapeutic use are generally delivered by encapsulation or adhesion to microspheres (He et al., 2020), and gene regulators are usually grafted onto the surface of prepared microspheres with the help of nanoparticles to achieve a mosaic effect (Feng et al., 2017). Moreover, the diverse fabrication processes yield microsphere products with different fine structures, such as porous, hollow, solid, and bilayer structures, which also give different medical uses to the microspheres.

**TABLE 1:** Introduction of several common fabrication methods of microspheres.

Microsphere Fabrication Methods	General Process	Merits	Drawbacks
Emulsion-solvent evaporation	Single emulsion method: The active substance and polymer material are dissolved in organic solvent to form the oil phase and then added to the aqueous phase containing emulsifier; after high-speed rotation to generate emulsion droplets, the organic solvent in the emulsion droplets is evaporated to obtain microspheres	Simple and easy to operate; a wider choice of materials; capable of encapsulating fat-soluble and water-soluble substances	Uneven size of microspheres; poor encapsulation of water-soluble substances; incomplete removal of organic solvents
	Double emulsion method: The active substance is dissolved in an aqueous medium to form an internal aqueous phase, followed by adding the internal aqueous phase to the oil phase containing the polymer to complete the primary emulsification. The primary emulsion is then added to the aqueous phase containing the emulsifier to complete the re-emulsification process. The microspheres are obtained by evaporation of the organic solvent		
Microfluidic technology	Immiscible liquid phases were injected into different microchannels to form microspheres under high shear stress	Relatively controllable particle size of microspheres; better reproducibility; aseptic	Lower production efficiency; difficulty cleaning equipment
Spray-drying	The prepared liquid of raw and auxiliary materials is atomized through the atomizing nozzle and then cured in the dry hot gas to form microspheres	High encapsulation rate and low loss of active substances; keeping substances active	Loss of raw materials; temperature seriously affects the quality of microspheres
Phase separation	The active substance is dissolved or dispersed in the organic phase dissolved with polymer, and then the organic nonsolvent is added to this organic phase to precipitate the polymer, thus encapsulating the active substance to form microdroplets. The microspheres are obtained after removing the excess components	Easy equipment and operation; high ball formation rate for water-soluble substances	Microspheres are agglomerated and not easily dispersed; easy residual organic solvents
Electrospray	The polymer solution containing the active substance is placed in the syringe of the electrospray device, and the droplets come out of the nozzle and are atomized microdroplets by voltage, collected at the bottom, dried, and cured to obtain microspheres	Microsphere size can be controlled by adjusting the voltage; higher encapsulation rate	Not much coverage

**FIGURE 5:**
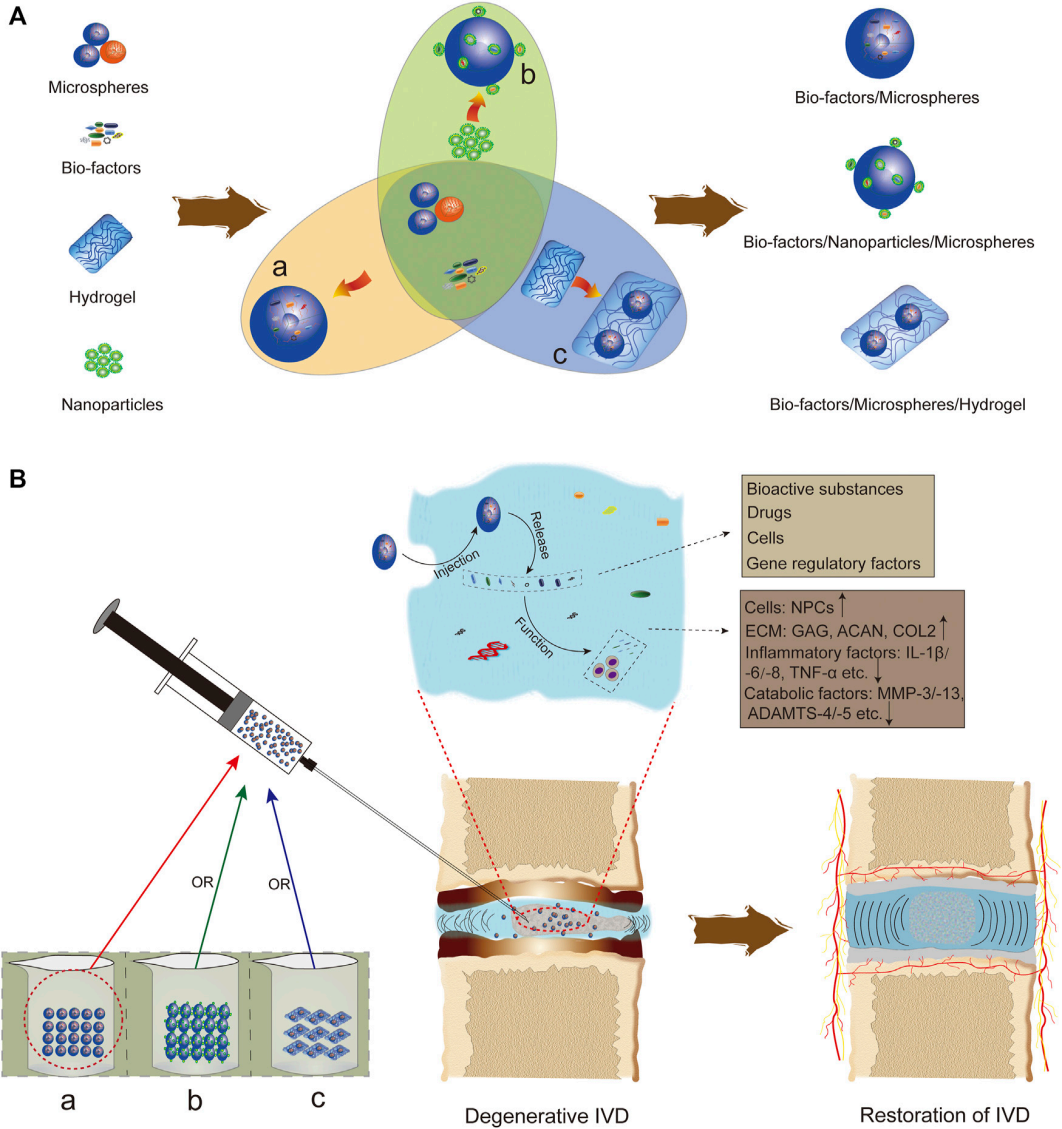
Application of microspheres in IVDD. **(A)** Currently, there are three forms of microsphere-based delivery systems for IVDD: **(a)** biofactors are loaded on microspheres by microencapsulation; **(b)** some biofactors are adhered to microspheres with the help of nanoparticles; and **(c)** microspheres are embedded in hydrogels after loading biofactors. **(B)** The microsphere delivery system plays a role through *in situ* injection into IVD tissue, including increasing IVD cells, promoting IVD matrix formation, and inhibiting the expression of inflammatory factors and catabolic mediators.

When degeneration occurs, the interior of the IVD presents a withering pattern with microscopic manifestations such as loss of ECM components, decreased number and activity of resident cells, and macroscopic changes such as decreased IVD height and water content and NP fibrosis (Milette et al., 1999; Gu et al., 2017), and this pathological change progressively worsens over time. As tissue engineering and regenerative medicine techniques continue to develop, microsphere-based regenerative therapies are gradually being used for IVDD, which has the opportunity to change the current dismal situation. In recent studies, microsphere systems carrying bioactive substances (Sawamura et al., 2009; Yuan et al., 2013; Yan et al., 2014; Xu H. et al., 2020; Xu Y. et al., 2020; Bian et al., 2021; Shen et al., 2022), drugs (Liang et al., 2013; Murab et al., 2015; Tellegen et al., 2018; Rudnik-Jansen et al., 2019; Zhu et al., 2020; Wiersema et al., 2021), cells (Liang et al., 2013; Li et al., 2014; Xia et al., 2019), and gene regulators (Feng et al., 2017; Feng et al., 2020; Chang et al., 2022) have been injected into IVD tissues in a minimally invasive manner to exert corresponding therapeutic effects locally (Figure 5). It is worth mentioning that therapeutic substances (except cells) are slowly released locally by self-diffusion, microsphere degradation, or dissolution. This microsphere delivery system targets tissue degeneration mechanisms and modulates them at the cellular, molecular and genetic levels in an attempt to curb IVDD progression, providing a pioneering strategy for the treatment of IVDD. In this section, microsphere delivery systems are discussed in terms of four aspects: targeted delivery of bioactive substances, local controlled release of drugs, cellular therapy, and gene therapy.

### 3.1 Targeted Delivery of Bioactive Substances

#### 3.1.1 Growth Factors

Growth differentiation factors (GDFs) belong to the transforming growth factor (TGF)-β superfamily, which can significantly affect the proliferation and differentiation of body cells and participate in the growth and development of tissues or organs. As an important member of this family, GDF-5 plays an important role in chondrocyte production and articular cartilage development, and its deletion leads to abnormal IVD matrix synthesis (Feng et al., 2015) and is considered a potential therapeutic target for degenerative joint diseases (Sun et al., 2021). Recently, several research teams tested the effects of GDF-5 delivery via microspheres for use in a rat IVDD model. The first group fabricated GDF5-PLGA microspheres by the W/O/W emulsion-solvent evaporation method. *In vitro* release experiments showed that the microsphere system achieved the release of active recombinant human (RH) GDF-5 over 42 days. Eight weeks after the treatment system was injected into the rat tail degeneration IVD model, imaging evidence showed that the GDF-5 sustained-release system effectively maintained the IVD height, and the detection of gene and protein levels also confirmed the increase in matrix expression (Yan et al., 2014). In another study, the authors prepared gelatin methacrylate (GelMA) microspheres (GMs) loaded with GDF-5 using an electrospray device. The obtained GDF5-GM was homogeneous in size, released GDF-5 continuously, and reached approximately 60% release after 25 days. Coculture with rat adipose-derived stem cells (ADSCs) *in vitro* showed that GDF5-GM possessed considerable biocompatibility and promoted the proliferation and NP-like differentiation of ADSCs. After implantation *in vivo*, GDF5-GM carrying ADSCs not only provided mechanical tension to degenerated IVD tissues but also sustained the release of GDF-5 to induce NP-like differentiation of stem cells, contributing to ECM synthesis and IVD height recovery (Xu H. et al., 2020). This finding suggests that the cell-growth factor-microsphere delivery system can effectively repair degenerated IVD tissues.

In addition, GDF-6 has a highly similar biological role to GDF-5, which induces bone marrow- and adipose-derived MSCs to exhibit an NP-like phenotype (Clarke et al., 2014) and promotes degenerated NPC regeneration (Hodgkinson et al., 2020). Hodgkinson et al. prepared copolymer (mainly PLGA) microparticles (MPs) loaded with rhGDF-6 by the W/O/W double emulsion technique and validated them *in vitro* (Hodgkinson et al., 2019). In 3D culture experiments with adipose stem cells (ASCs), GDF6-MP ensured a continuous supply of active rhGDF-6, which effectively induced ASC differentiation to NPC and promoted the secretion of sulfated glycosaminoglycan (GAG) and ACAN. It follows that the use of microspheres for the slow release of GDF-5 and GDF-6 has the potential to promote IVD regeneration, but the simultaneous delivery of cells and growth factors has significantly more potential.

TGF-β3 belongs to the TGF-β subtype, which regulates cell proliferation, differentiation, and migration and plays an important role in the growth and repair of cartilage (Yoo et al., 2022). Currently, TGF-β3 has been used as a minimally invasive therapy for IVDD. Tsaryk and coworkers developed an NP-like tissue engineering material by mosaicking gelatin microspheres loaded with TGF-β3 in a polymer network composed of COL and low molecular weight hyaluronic acid (HA) (Tsaryk et al., 2015). *In vitro* experiments showed that both TGF-β3-microspheres and COL-HA hydrogels could provide a suitable microenvironment for chondrogenic differentiation of MSCs and nasal chondrocytes, and the release of TGF-β3 from microspheres showed a time-dependent effect. Subsequent subcutaneous transplantation of NP-like material carrying cells confirmed the deposition of COL2 and GAG. This cell-microsphere-hydrogel system may hold promise as an NP alternative and provide a feasible strategy for IVD regeneration.

#### 3.1.2 Inflammatory Antagonists

Inflammation has a nonnegligible role in the development of IVDD, and IL-1β and TNF-α play important roles in the inflammatory cascade response (Bian et al., 2021). Therefore, antagonist therapies targeting key inflammatory mediators have been attempted for the treatment of IVDD. IL-1 receptor antagonists (IL-1ra) are rightfully the focus, but their systemic administration or repeated local injections may outweigh the benefits. To this end, Gorth and others synthesized IL1ra-PLGA microspheres using PLGA (75:25, 0.1 mg/ml) and IL-1ra by a double emulsification technique and characterized and simulated their applications (Gorth et al., 2012). The results showed that the IL1ra-PLGA microspheres were porous, with a diameter of 5–20 μm, and could be released cumulatively for more than 35 days. In the culture system of NP constructs, the sustained release of IL-1ra mediated by microspheres could comprehensively limit the degradation (mainly in terms of mechanical properties, GAG content, and catabolic factor mRNA levels) of the constructs by IL-1β within 1 week, but this limitation became progressively weaker as IL-1ra activity and release decreased. Thus, IL1ra-PLGA microspheres successfully attenuated the damaging effects of IL-1β-induced inflammation and may treat inflammation-mediated early degeneration of IVD.

Similarly, recombinant human soluble TNF receptor II (rhsTNFRII) attenuates the inflammatory response of IVD tissues by inhibiting the activity of TNF-α. Scholars grafted rhsTNFRII-encapsulated bovine serum albumin nanoparticles onto PLLA microspheres prepared by microfluidic and porogenic techniques to construct an injectable antagonist formulation (Xu Y. et al., 2020). The porous structure of PLLA microspheres ensures a uniform and sustained release of rhsTNFRII with an overall release rate of 79.85 ± 2.12% over 35 days. Significant inhibition of the expression of inflammatory factors such as TNF-α and MMP-3 was observed with *in vitro* cellular studies, while the expression of COL2 and metalloproteinase tissue inhibitor-1 was enhanced. On the other hand, intra-IVD injections of functionalized antagonists also reaped exciting results, with a higher IVD height index (DHI) and matrix (COL2 and PG) content obtained in the rhsTNFRII-microsphere treatment group (Xu Y. et al., 2020). This revealed that functionalized formulations composed of antagonists and microspheres could restore ECM metabolic homeostasis by modulating the inflammatory microenvironment and achieving NP tissue regeneration.

Aberrant activation of acid-sensitive ion channel 3 (ASIC-3) under low pH conditions in IVDD has been reported to trigger pain and inflammatory factor expression in NPC (Gilbert et al., 2016). Correspondingly, the sea anemone peptide APETx2 could specifically inhibit ASIC-3 activation and prevent the inflammatory response in NP tissues. Bian and his colleagues constructed an injectable “cell-peptide-microsphere” delivery system by covalently grafting APETx2 on GelMA microspheres and seeding it with NPC and tested it in both *in vitro* and *in vivo* settings (Bian et al., 2021). They detected that the peptide-microspheres sustained the release of APETx2 over 28 days in an acidic environment *in vitro*. *In vitro* coculture of NPC and peptide-loaded microspheres revealed that ASIC-3 overexpression under acidic conditions was significantly inhibited by APETx2 and that the expression of inflammatory or catabolic factors such as IL-1β, TNF-α, MMP-3 was downregulated, while COL2 and ACAN appeared to be highly expressed. In addition, an 8-weeks *in vivo* experiment showed that the cell-peptide-microsphere treatment group was closer to normal in terms of DHI and ECM deposition than the other groups (Bian et al., 2021). Thus, APETx2 did not affect the biocompatibility of microspheres, and the APETx2-NPC-microsphere delivery system could promote the production of ECM components by inhibiting NP hyperinflammation, exhibiting a strong IVD regenerative potential.

#### 3.1.3 Nanoenzyme

Because there is no blood supply in NP tissue and the central region is a microenvironment of low oxygen and glucose, a considerable part of the tissue cell’s energy is derived from anaerobic glycolysis, which can cause lactic acid accumulation. However, lactate is closely associated with inflammatory immune responses (Certo et al., 2021), and excess lactate will be accompanied by apoptosis of NPC and low expression of ECM components (Shi et al., 2019) and can lead to IVD-derived back pain (Keshari et al., 2008). Therefore, the removal of excess lactate within the NP may inhibit IVD inflammation and improve the cell survival microenvironment. In a recent study, lactate oxidase (LOX) was electrostatically bound to a presynthesized MnO_2_-chitosan “shell nucleus” structure to form a MnO_2_-chitosan-LOX (MCL) nanoenzyme, which was subsequently coupled to methacrylated hyaluronic acid (HAMA) microspheres and evaluated (Shen et al., 2022). Importantly, LOX in the MCL nanoenzyme oxidizes lactate to pyruvate and hydrogen peroxide (H_2_O_2_), while MnO_2_ reacts with H_2_O_2_ to generate oxygen, avoiding possible oxidative stress and facilitating the catalytic reaction of LOX. The results of cellular experiments showed that in the lactic acid environment, the MCL-microsphere group had high survival of NPC with higher levels of ACAN and COL2 expression and downregulated expression of genes for inflammatory factors (IL-1β and TNF-α) and catabolic factors (e.g., MMP-3, MMP-13). Likewise, the MCL-microsphere group performed well in maintaining IVD height and ECM deposition and controlling the expression of inflammatory factors in a lactate-induced degenerative IVD model in rats (Shen et al., 2022). Therefore, injectable nanoenzyme-functionalized microspheres may improve the microenvironment for IVD cell survival by consistently alleviating lactate accumulation and may inhibit tissue inflammation and promote matrix synthesis, providing some guidance for IVD regeneration.

#### 3.1.4 Platelet Rich Plasma (PRP)

PRP is the product of whole blood centrifugation and is of interest because it is rich in growth factors such as TGF-β, platelet-derived growth factor, basic fibroblast growth factor, and insulin-like growth factor 1 (Chang et al., 2020). Based on this property, PRP has been widely used for IVDD treatment in the last decade. Numerous cellular and animal experiments have provided sufficient theoretical justification for the use of PRP for IVD regeneration, and even direct injection of PRP in human IVD can effectively alleviate LBP symptoms (Chang et al., 2020). However, direct PRP injections have only short-lived efficacy, whereas sustained release via microspheres has unexpected effects. Researchers developed a PRP-loaded polyethylene glycol (PEG) microsphere (∼100 µm diameter and ∼99% loading efficiency) and characterized the degradation and release of two microspheres loaded with 10% w/v powder PRP and liquid (micro agglomerate removed) PRP (Choi et al., 2020). Subsequent results showed that PRP could prolong the degradation and protein release time of microspheres, but the effect of powdered PRP microspheres on delaying degradation and release due to coagulation was more significant. This finding reveals that the presence of PRP can effectively prolong the treatment of microspheres, which is significant for repairing chronic damage in degenerative diseases. Sawamura et al. formed PRP microspheres by impregnating PRP into gelatin hydrogel microspheres and used them in a degenerative IVD model in rabbits, which showed good tissue repair (Sawamura et al., 2009). These studies have confirmed the prospect of PRP for IVD regeneration through the slow release of microspheres.

#### 3.1.5Decellularized NP Matrix (DNPM)

Artificially constructed biomaterial scaffolds, despite having been desperately tried to create a microenvironment and biomechanical structure close to that of natural tissues, still have many shortcomings, such as lacking the well-established cellular niche, 3D microstructure, and growth factor environment possessed by natural matrices (Xu J. et al., 2019). In contrast, decellularized tissue matrix retains various beneficial components of the native matrix and is tissue-specific (Peng et al., 2021). For example, DNPM better eliminates immunogenicity through a decellularization process, possesses a matrix niche for inducing stem cell growth and directed differentiation, and can serve as a potential bionic scaffold in the field of NP regeneration. It is undeniably an interesting experience to construct DNPM microcapsules and perform a 3D culture of cells and use them for IVDD models. Yuan and coworkers successfully constructed COL microspheres retaining NPC-derived matrix by microencapsulation and decellularization techniques, and further exploration was carried out after inoculation of human and rabbit MSCs on this microsphere (Yuan et al., 2013). The authors have determined the presence of GAG, COL2, keratin 19, TGF-β, and their receptors in the obtained DNPM. Eighteen days of *in vitro* coculture showed that hMSCs survived and highly expressed genes for human NPC-specific markers such as COL2A1, glypican 3 and forkhead box F1 in DNPM-COL microspheres. This finding suggests that DNPM induces the differentiation of hMSCs into NPCs. On the other hand, infusion of rMSC-DNPM microspheres in rabbit degenerative IVD maintained high COL2 expression and hydration index; unfortunately, there was not much improvement in IVD height (Yuan et al., 2013). This may be related to the fact that decellularization disrupts the fine structure of the matrix and weakens the mechanical properties of the microspheres. In conclusion, DNPM microspheres provide a near-natural matrix niche for the growth, proliferation, and NP-like differentiation of MSCs, adding potential promise for IVD regenerative therapies.

### 3.2 Drug Delivery and Controlled Release

#### 3.2.1 NSAIDs

Although NSAIDs are quite commonly used in the management of chronic LBP in IVDD, high-dose systemic administration may not necessarily achieve local therapeutic concentrations due to the specificity of the IVD blood supply (limited to the outer AF). Therefore, local health problems similar to IVDD and osteoarthritis (OA) necessitate the consideration of topical medications. In particular, unlike traditional nonselective NSAIDs, celecoxib (CXB), the world’s first selective cyclooxygenase-2 (COX-2) inhibitor, has a favorable safety profile and therapeutic potential (Yeomans et al., 2018). Currently, CXB-based topical drug delivery systems have been widely used for the treatment of IVDD and OA. A study in a canine IVDD model explored the efficacy of a CXB delivery and sustained-release system in repairing degenerated tissue, anti-inflammatory, and anti-matrix degradation (Tellegen et al., 2018). This research team prepared two types of poly(ester amide) (PEA) microspheres with low and high CXB loading by emulsion solvent evaporation and observed that CXB achieved sustained release on the microspheres for at least 28 days under inflammatory conditions. *In vitro* experiments showed that CXB inhibited the production of prostaglandin E_2_ (PGE_2_) in NPC and significantly downregulated the mRNA expression of MMP-13 and a disintegrin and metalloproteinase with thrombospondin motifs 5 (ADAMTS-5) in NPC cells. This revealed sustained anti-inflammatory and anti-catabolic effects of the CXB-PEA microspheres. In addition, high expression of ACAN and COL2A1 confirmed the propensity for matrix deposition, and interestingly, these effects were more pronounced in the high CXB dose (10^−4^ M) microsphere culture system, suggesting a dose-dependent physiological activity of CXB. Furthermore, CXB-PEA microspheres have shown promising efficacy in degenerative IVD by inhibiting the production of PGE_2_ and nerve growth factor (NGF) and effectively avoiding the loss of IVD substances such as water and GAG (Tellegen et al., 2018). This suggests that the CXB-PEA microsphere slow-release system may reduce inflammation and IVD-derived pain in the long term and, importantly, has the potential for structural repair while inhibiting degeneration. In the future, human trials are necessary to verify the clinical efficacy of CXB-PEA microspheres. Unfortunately, microsphere delivery systems of other NSAIDs for IVDD have yet to be further developed.

#### 3.2.2 Corticosteroids

Corticosteroids are widely used clinically as anti-inflammatory drugs and immunosuppressants, and their local injections are commonly used to relieve inflammatory pain caused by IVDD and OA. However, local injections of corticosteroids may pose serious risks due to systemic exposure and have short maintenance of efficacy, often requiring repeated injections (Tryfonidou et al., 2020). Therefore, how to effectively prolong the local action time of drugs has always been the direction of researchers, and formulating drugs into microspheres is a promising strategy.

The use of triamcinolone acetonide (TA) in combination with microspheres has been a research hotspot in IVDD and OA, and in particular, TA-PLGA microspheres (trade name: FX006) have been used in clinical trials for long-term analgesia in patients with OA (Conaghan et al., 2018). Recently, Rudnik-Jansen et al. evaluated the safety and benefits of TA-loaded PEA microspheres for use in canine animals, where they induced an IVDD model at the lumbar spine level by partial removal of NP tissue and performed TA-PEA microsphere implantation after 4 weeks (Rudnik-Jansen et al., 2019). All dogs used in the test were free of other diseases after treatment, demonstrating the safety of TA-PEA microspheres *in vivo*. Additionally, NGF expression associated with IVD-derived LBP and inflammation was suppressed in NP tissues, indicating a positive effect of the TA slow-release system on pain relief and anti-inflammation. Nevertheless, there was no significant improvement in IVD structure and integrity at either macroscopic (e.g., imaging presentation) or microscopic (ECM expression) levels after TA-PEA microsphere treatment. In summary, TA-PEA microspheres may provide continued pain relief for IVDD patients by inhibiting inflammation but are not beneficial in preventing disease progression.

Furthermore, dexamethasone (DEX) is a potent anti-inflammatory agent for tissues, although its high therapeutic dose can lead to many side effects (Urbańska et al., 2014). Thus, the release of DEX via microspheres is attractive for alleviating local inflammation. Scholars have explored the potential of the dual delivery system of microspheres loaded with DEX and growth factors in inducing stem cells to differentiate into NPCs. Liang and coworkers successively loaded DEX and basic fibroblast growth factor (bFGF) on PLGA microspheres and cultured MSCs *in vitro* (Liang et al., 2012). Subsequently, they found that DEX and bFGF release from the microspheres did not interfere with each other and that MSCs in the system expressed an IVD matrix. The composite microsphere system supports the growth and NP-like differentiation of stem cells, especially DEX, which can avoid the influence of the inflammatory environment on cells. Soon after, the group used a PLGA microsphere system loaded with DEX, TGF-β3, and ADSCs in a rat IVDD model, resulting in significant recovery of IVD height and matrix content in the DEX-TGFβ3-ADSC microsphere treatment group (Liang et al., 2013). Therefore, we know that this delivery system of stem cells and microspheres at least partially promotes IVD regeneration, but it is worth noting that it is equally important for DEX to eliminate implant-induced inflammation.

#### 3.2.3 Other Drugs

Although NSAIDs and corticosteroids achieve localized delivery and sustained release through microspheres, their overall role in IVDD is limited to the control of inflammation and relief of LBP symptoms due to the drugs themselves, and they are not as effective in IVD repair or regeneration. Even so, more promising drugs are being discovered and used for IVD reconstruction by microencapsulation.

Glucosamine is an essential component for the synthesis of glycoproteins and GAGs in the cartilage matrix and is widely used in the United States as a dietary supplement for cartilage health care (Henrotin et al., 2014). Due to the similarity in matrix composition between IVD and articular cartilage, N-acetyl-glucosamine (GlcNAc), a derivative of glucosamine, has long been explored for IVD regeneration. Murab et al. produced an injectable silk hydrogel embedded with GlcNAc-loaded silk microspheres and used it in an *ex vivo* IVDD model (Murab et al., 2015). They prepared silk protein hollow microspheres by a template method that allows controlled release of GlcNAc in the hydrogel system to support the growth and differentiation of human ADSCs. Cellular experiments revealed increased expression of GAG, COL2, and ACAN in ADSCs. Similarly, significant ECM accumulation and elevated compressive strength of IVD tissues were detectable after injection of the composite system into a degraded IVD model. It is known that the microsphere-hydrogel chimeric system improves the mechanical properties of IVD due to its structure and matrix deposition effect, which may be a potential strategy for IVD regeneration and deserves to be explored in depth.

Epigallocatechin 3-gallate (EGCG) is a polyphenol derived from tea tree that exerts IVD protective effects through anti-inflammatory and antioxidant mechanisms (Krupkova et al., 2014; Krupkova et al., 2016b). However, the stability of EGCG is susceptible to its environment (enzymes, temperature, pH), and therefore it is difficult to maintain high biological activity (Krupkova et al., 2016a). Based on this concern, a team produced large quantities of gelatin microspheres loaded with EGCG by a modified electrospray technique and systematically validated them *in vitro* (Loepfe et al., 2019). Coculture with IVD cells in 3D alginate constructs showed that EGCG-GM had excellent biocompatibility and significantly inhibited the expression of IL-1β-induced inflammatory factors (IL-6, IL-8, and COX-2) and catabolic mediators (MMP-1, -3, and -13). This confirmed that the EGCG released from the microspheres maintained sufficient activity and anti-inflammatory capacity. If used for degenerated IVD tissue, the sustained-release system may prevent it from being damaged by inflammation, which has certain therapeutic significance.

There are many types of statins, including but not limited to simvastatin, lovastatin, and rosuvastatin. They are commonly used to treat hypercholesterolemia, but other therapeutic roles are also broad. For example, the application of simvastatin within a degenerative IVD may slow the degenerative process to some extent (Than et al., 2014). One study reported the effect of simvastatin-loaded PLGA microspheres in a rat model of IVDD (Zhu et al., 2020). Simvastatin microspheres showed a better release profile, with a cumulative release of more than 80%. After 4 weeks of *in vivo* treatment, the simvastatin microsphere-treated group reconstructed the bone mineral density of the corresponding vertebral body, and the T2-weighted signal of NP tissue MRI was close to the physiological level, indicating an improvement in tissue degeneration. In brief, microsphere delivery therapy with statins may represent a promising strategy for IVD regeneration.

### 3.3 For Cell Production, Storage, and Transplantation

#### 3.3.1 Customization, 3D Culture, and Storage of Cells

Prior to cell transplantation, the acquisition, culture, and preservation of therapeutic cells are issues that need to be properly addressed. First, the microsphere system can be used as a functionalized platform for producing cells with IVD regenerative potential. To this end, Fontana et al. designed a 3D microgel system composed of COL2 and hyaluronic acid containing ADSCs and transfectable pDNA for the construction of NP-tailored cell factories (Fontana et al., 2015). This functionalized microgel system allows for targeted modification of stem cells for the expression of specific proteins and may even provide a reliable source of cells for cellular therapies that promote NP regeneration. Second, the 3D microsphere system provides a more refined biomimetic microenvironment for all biological behaviors of cells. Compared to conventional two-dimensional culture systems, 3D gelatin microsphere systems support higher levels of cell activity and proliferation and can effectively maintain cell morphology and phenotypic secretion (Sulaiman et al., 2020). This confirms the great potential of the microsphere system for cell customization and culture. Finally, ideal cell therapy places many requirements on cells for transplantation, such as the ability to direct differentiation, the generation of specific phenotypes, and the convenience of “ready access.” Directed differentiation and specific expression of seed cells are often achieved by adding growth factors, whereas “ready access” requires advanced stocking of cells. Naqvi and his colleagues developed a cell pretreatment and freezing strategy for IVD regeneration (Naqvi et al., 2018). After encapsulation of bone marrow stromal cells (BMSCs) in alginate microcapsules, microencapsulated BMSCs were induced to differentiate by applying 14 days of TGF-β3 pretreatment and 21 days of reculture with or without growth factors, and COL and sulfated GAG appeared to be abundantly expressed. Notably, cryopreserved BMSCs also exhibited robust ECM-producing capacity in 21-day recultures. Similar results were obtained using BMSC microcapsules in an *in vitro* coculture model and *ex vivo* oxtail IVD (Naqvi et al., 2019). Thus, pretreatment with BMSC microcapsules can induce the differentiation of BMSCs and initiate the IVD-like phenotype. More importantly, the cryopreservation of BMSC microcapsules does not affect the biological activity of BMSCs, which can effectively alleviate the challenges associated with cell storage and transport, allowing for more flexible cell therapies for IVD repair.

#### 3.3.2 Cell Transplantation

In recent years, polymeric microspheres have often been used as delivery vehicles for cells due to their nontoxic, biocompatible, and biodegradable properties and have great potential for cell therapy in IVD tissue engineering. A team tested the effectiveness of microspheres as cell carriers in a rat IVD model. In their study, gelatin microspheres containing GDF-5 were prepared by the W/O emulsion method, and the differentiation of induced pluripotent stem cells (iPSCs) to NP-like cells (NP-LCs) was achieved in two steps, followed by confirmation that NP-LCs could adhere to GDF5-GM (Xia et al., 2019). After 24 weeks of *in vivo* treatment, both the DHI and IVD water contents in the NP-LCs/GDF5-GM group were closest to those in the negative control group and fared better than those in the other groups. In addition, IVD tissue staining in this treatment group also confirmed more COL2 deposition. To a certain extent, cell replacement therapy with microspheres as carriers can effectively replenish IVD cells, partially restore IVD height and ECM components and have a considerable effect on IVD regeneration. Moreover, although cell implantation is performed by minimally invasive *in situ* injection, the leakage of seed cells and materials cannot be completely avoided, which will eventually lead to complications such as osteophytes. However, the use of microspheres as cell carriers may improve this situation. Li et al. encapsulated MSCs in porous collagen microspheres to form MSC microcapsules and validated the effect of MSC-containing saline and collagen microspheres after injection into IVDs (Li et al., 2014). Compared with MSCs in the saline group, MSCs in the microsphere group were better able to maintain the dynamic mechanical properties of the spinal motion segments and significantly reduce osteophyte formation at 6 months, although they did not have an advantage in maintaining DHI and hydration index. It follows that the use of cell transplantation for the treatment of IVDD should focus on the selection of cell carriers, and the selection of suitable microsphere transport carriers will greatly avoid complications while ensuring the therapeutic effect.

### 3.4 Gene Therapy

Gene therapy is an emerging therapeutic strategy for genetically related diseases that have long been widely studied. It generally treats diseases by introducing exogenous DNA or RNA into specific cells with the help of transfer technology to improve their expression. Gene transfer technology has undergone a long evolution from direct injection to viral vectors and then to nonviral vectors. However, thus far, the development of safe and efficient nucleic acid carriers remains a key challenge for gene therapy. Currently, viral gene vectors include adenoviruses, adeno-associated viruses, lentiviruses, and retroviruses (Sayed et al., 2022). Although viral vectors have made breakthroughs in the field of gene therapy, their oncogenicity, immunogenicity, and toxicity also severely limit their clinical applications (Kaiser, 2020). In recent years, research on nonviral gene delivery has become popular. From the nanoscale (e.g., liposomes (Wang et al., 2021; Yu et al., 2022), complex micelles (Yoshinaga et al., 2021; Yoshinaga et al., 2022), and exosomes (Cui et al., 2022; Xu et al., 2022)) to the micron scale (such as microspheres (McMillan et al., 2018; Hinkelmann et al., 2022)) or combined forms of nanoparticles and microspheres (Feng et al., 2017; Feng et al., 2020; Chang et al., 2022), more options for nonviral vectors have been provided. Importantly, genetic factors contribute to the worsening of IVDD, and gene therapy approaches are expected to improve the poor genetic cascade regulation of IVD. Recently, a combination strategy of nanoparticles and microspheres has started to be used for gene delivery therapy of IVDD, which can accomplish the genetic modification process with precision and efficiency.

#### 3.4.1 Nuclear Receptor 4A1 (NR4A1) Plasmid DNA (pDNA) and Anti-miR-199a

NP fibrosis is an important manifestation of IVDD, and inhibition of the fibrotic process contributes to the treatment of degenerative IVD (Kong et al., 2021). Correspondingly, small-molecule NR4A1 agonists inhibit fibrosis in multiple organs in experimental mice; thus, NR4A1 is considered a potential therapeutic target for fibrotic diseases (Palumbo-Zerr et al., 2015). Local transfection of genes in IVD to promote NR4A1 expression may be an effective way to inhibit fibrosis in IVD tissues. For this purpose, Feng and coworkers combined nanospheres (NS) and microspheres to enable the delivery and transfection of pDNA encoding NR4A1 (Feng et al., 2017). They encapsulated a complex formed by pDNA with hyperbranched polymer (HP) in PLGA NSs and then injected a mixture of pDNA/NS combined with porous nanofibrous spongy microspheres (NF-SMS) into the rat tail IVD. In this study, after the mixture of pDNA/NS and microspheres entered IVD, the pDNA complex was released along with the degradation of NS, which made pDNA transfect NPC at high speed, thereby overexpressing NR4A1. *In vitro* results showed that overexpression of NR4A1 significantly inhibited TGF-β1-induced fibrosis. Likewise, *in vivo* experiments confirmed that the NR4A1 pDNA-treated group had more IVD stromal deposition (high GAG content) and less fibrous tissue infiltration than the untreated group (no pDNA) with highly reduced IVD and extensive tissue fibrosis (Feng et al., 2017). It was shown that this microsphere-based delivery system can upregulate NR4A1 expression through pDNA transfection as a way to inhibit NP tissue fibrosis, maintain IVD biological function, and provide the possibility of IVD regeneration. Several years later, the team conducted another study using the same delivery system in which they loaded anti-miR-199a (AMO) on HP and inoculated MSCs with NF-SMS to achieve sustained release of AMO and efficient transfection of MSCs (Feng et al., 2020). The results from *in vitro* and subcutaneous studies in nude mice revealed that the sustained action of AMO promotes hypoxia-inducible factor-1α expression by inhibiting miR-199a, thereby inducing NP-like differentiation and preventing osteogenic differentiation in MSCs. In the rabbit IVDD model, the MSC/AMO microsphere treatment group showed a DHI and matrix content closer to normal levels and a much smaller volume of heterotopic ossification than the MSC microsphere treatment and sham groups, demonstrating good resistance to the calcification (Feng et al., 2020). It can be concluded that the slow release of anti-miR-199a on microsphere systems can provide effective genetic modification of MSCs to differentiate toward NP while blocking the tendency of ossification, which is of great significance for NP tissue regeneration and prevention of IVD calcification.

#### 3.4.2 CircSTC2 Silencing Genes

Although the etiological mechanisms of IVDD development have not been elucidated, certain circRNAs present in NP tissues are aberrantly expressed under the harsh conditions of IVD tissue degeneration and nutrient deficiency (Chang et al., 2021). This can cause disturbances in ECM anabolism and catabolism, but targeted regulation of circRNAs has the potential to prevent abnormal metabolism and thus restore ECM homeostasis. Chang et al. pioneered the use of cationic liposomes to load silencing genes and graft them onto HAMA microspheres and subsequently tested the effects of this liposome-microsphere system (circSTC2/lipo/MS) for circRNA silencing in nutrient-limited *in vivo* and *in vitro* environments (Chang et al., 2022). The authors used a microfluidic device to fabricate porous HAMA microspheres (257.86 ± 13.45 µm) and detected the encapsulation efficiency (95.60%) and drug loading efficiency (2.05%) of the lipoplex for pDNA. The release experiment showed that the microsphere delivery system equipped with pDNA-liposomes could be released continuously for 27 days, and the total release amount was more than 80%. *In vitro* culture of NPC revealed a significant upregulation of ACAN and COL2 in the circSTC2/lipo/MS group compared to the glucose-free group. Even the expression level of COL2 exceeded that of the normal culture group at both the gene and protein levels. In the nutrient-deficient rat IVD model, the circSTC2/lipo/MS treatment group also exhibited satisfactory effects. After 8 weeks of treatment, IVD had a highly significant recovery, with better IVD architecture and more ECM deposition observed on histological staining (Chang et al., 2022). Therefore, the circSTC2/lipo/MS delivery system can silence circSTC2 in NPC, maintain ECM metabolic homeostasis in a nutrient-deficient environment, better remodel the IVD morphological structure, and possess good repair effects on degenerated IVD tissues.

In summary, microsphere delivery systems have been widely used to relieve the pain of IVD origin and to regenerate or repair IVD tissue. Although these studies are still in the preclinical stage, the use of microspheres as delivery vehicles for active substances, drugs, cells, and gene regulators for the treatment of IVDD has yielded exciting efficacy (Table 2). Shortly, this microsphere therapy may be an effective alternative to the current treatment option, bringing good news to more IVDD patients.

**TABLE 2:** Application of microsphere-based delivery systems in IVDD.

Components of the Delivery System	Fabrication Method	Substance Delivered	Experiments	Function	Ref
PLGA microspheres	W/O/W double emulsion method	rhGDF-5	Rat IVDD model	Restoration of IVD heights; promote IVD matrix expression	Yan et al. (2014)
GelMA microspheres	Electrospray	GDF-5; ADSCs	Cell; rat IVDD model	Promotion of ADSC proliferation and NP-like differentiation; restoration of IVD heights; promote the synthesis of ECM	Xu H. et al. (2020)
Copolymer microparticles	W/O/W double emulsion method	rhGDF-6	Cell	Induction of ACSs to NPC differentiation; promote secretion of sulfated GAG and ACAN	Hodgkinson et al. (2019)
Gelatin microspheres and collagen-LMW HA hydrogel	Microspheres: unclear; hydrogel: gelation	TGF-β3	Cell; subcutaneously implanted into female SCID mice	Promoting chondrogenic differentiation of MSCs and nasal chondrocytes; promote the production of COL2 and GAG	Tsaryk et al. (2015)
PLGA microspheres	W/O/W double emulsion method	IL-1ra	Cell; NP constructs	Inflammatory inhibition; attenuate the degradation of NP constructs by IL-1β	Gorth et al. (2012)
PLLA microspheres	Microfluidic technology	rhsTNFRII	Cell; rat IVDD model	Effectively inhibit IVD inflammation; maintain IVD height and matrix content	Xu Y. et al. (2020)
GelMA microspheres	Microfluidic technology	APETx2; NPC	Cell; rat IVDD model	Inhibit the expression of IL-1β, IL-6, TNF-α, MMP-3 and ADAMATs-5; promote the expression of COL2 and ACAN; inhibition of degenerative processes	Bian et al. (2021)
HAMA microspheres	Microfluidic technology	Lactate oxidase enzyme	Cell; rat IVDD model	Inhibition of inflammation and lactic acid accumulation; maintain a high level of IVD; promote IVD matrix expression	Shen et al. (2022)
Gelatin hydrogel microspheres	Gelation	PRP	Rabbit IVDD model	Inhibition of NPC apoptosis; promote the mRNA expression of COL2 and PG	Sawamura et al. (2009)
Collagen microspheres	Gelation	DNPM; MSCs	Cell; rabbit IVDD model	Induction of differentiation of MSC to NPC; promote GAG and COL2 production	Yuan et al. (2013)
PEA microspheres	Emulsification method	Celecoxib	Cell; canine IVDD model	Inhibition of PGE2 and NGF expression; anti-inflammatory and anti-catabolic; relief of IVD-derived pain; promote ECM synthesis	Tellegen et al. (2018)
PEA microspheres	Emulsification method	Triamcinolone acetonide	Dog IVDD model	Inhibition of NGF expression; relief of pain symptoms	Rudnik-Jansen et al. (2019)
PLGA microspheres	Emulsion solvent evaporation method	Dexamethasone; TGF-β3; ADSCs	Rat IVDD model	Significant restoration of IVD height and matrix content	Liang et al. (2013)
PLGA microspheres	Emulsion solvent evaporation method	Dexamethasone; bFGF; MSCs	Cell	Promote the expression of IVD matrix components; inhibits inflammation	Liang et al. (2012)
Silk microspheres and silk hydrogel	Microspheres: template method	GlcNAc	Cell; bovine *ex-vivo* IVDD model	Promote the expression of GAG, COL2 and ACAN; enhanced the mechanical properties of the *ex-vivo* IVD model	Murab et al. (2015)
Gelatin microspheres	Electrospray technology	EGCG	Cell	Inhibition of the expression of inflammatory factors and catabolic mediators	Loepfe et al. (2019)
PLGA microspheres	Emulsification method	Simvastatin	Rat IVDD model	Increase the vertebral body bone density; promote matrix deposition in NP	Zhu et al. (2020)
Gelatin microspheres	W/O emulsion method	NP-LCs; GDF-5	Rat IVDD model	Recovery of IVD height and water content; promote ECM synthesis	Xia et al. (2019)
Collagen microspheres	Gelation	BMSCs	Rabbit IVDD model	Partial promotion of NP matrix synthesis; maintenance of dynamic mechanical properties of spinal motor segments; reduce osteophyte formation	Li et al. (2014)
Nanofibrous spongy microspheres and PLGA nanospheres	Microspheres: unclear; nanospheres: W/O/W double emulsion method	NR4A1 pDNA	Cell; rat IVDD model	Inhibition of NP tissue fibrosis; promotion of GAG production; partial restoration of IVD height	Feng et al. (2017)
Nanofibrous spongy microspheres and PLGA nanospheres	Microspheres: phase-separation method; nanospheres: W/O/W double emulsion method	Anti-miR-199a; BMSCs	Cell; subcutaneous implantation in nude mice; rabbit IVDD model	Promoting NP-like differentiation of MSC; effective maintenance of IVD height; inhibition of IVD tissue calcification	Feng et al. (2020)
HAMA microspheres and cationic liposomes	Microspheres: microfluidic technology; Liposomes: film dispersion method	CircSTC2 silencing genes	Cell; rat IVDD model	Promote the synthesis of COL2 and ACAN; inhibit the expression of ADAMTS-4 and MMP-13; IVD height and structure recovered significantly; restoring ECM metabolic homeostasis	Chang et al. (2022)

PLGA, poly(lactic-co-glycolic acid); PLLA, poly-l-lactic acid; GelMA, gelatin methacryloyl; PEA, poly(ester amide); HAMA, methacrylated hyaluronic acid; W/O/W, water-oil-water; rhGDF, human recombinant growth differentiation factor; TGF, transforming growth factor; IL-1ra, interleukin-1, receptor antagonist; rhsTNFRII, recombinant human soluble tumor necrosis factor receptor II; PRP, platelet-rich plasma; DNPM, decellularized nucleus pulposus matrix; bFGF, basic fibroblast growth factor; GlcNAc, N-acetyl-glucosamine; EGCG, epigallocatechin 3-gallate; NR4A1, nuclear receptor 4A1; 6-K-PGF1α, 6-keto-prostaglandin F1α; HIF-1α, hypoxia inducible factor-1α; PGE_2_, prostaglandin E_2_; NGF, nerve growth factor; SCID, severe complete immunodeficiency; NPC, nucleus pulposus cell; MSCs, mesenchymal stem cells; BMSCs, bone marrow mesenchymal stem cells; NP-LCs, nucleus pulposus like cells; ADSCs, adipose-derived stem cells; IVDD, intervertebral disc degeneration; COL2, type II, collagen; ACAN, aggrecan; PG, proteoglycan; GAG, glycosaminoglycan; ADAMTS, a disintegrin and metalloproteinase with thrombospondin motifs; MMP, matrix metalloproteinase; ECM, extracellular matrix.

## 4 Clinical Transformation and Challenges of Microspheres

As a common class of drug carriers, microspheres are being developed on a large scale and have high expectations in serving clinical purposes. The microspheres that carry the hope must possess excellent properties (such as suitable biocompatibility, degradability, and mechanical properties, as well as a certain drug encapsulation rate and drug delivery efficiency) to ensure high functionality and low biologic aggression. The U.S. Food and Drug Administration (FDA) has a positive binding effect on these scaffolds and drug products for tissue use (Gottlieb, 2017; Naci et al., 2017). In the last decade, drug delivery technology for microspheres has developed rapidly and clinical trials related to it have been widely conducted worldwide. Microsphere delivery systems have shown exciting potential for application in humans, mainly focused on the control and treatment of several diseases, including cancer (Vilgrain et al., 2017; Chow et al., 2018; Ricke et al., 2019; Edeline et al., 2020; Boas et al., 2021; Garin et al., 2021), diabetes (Bode et al., 2015; Rosenstock et al., 2015; Seaquist et al., 2020), and osteoarthritis (Conaghan et al., 2018; Kraus et al., 2018). We can fully expect breakthrough efficacy and advances in these diseases from microsphere formulations. Notably, microsphere delivery and embolization strategies are research hotspots in oncology therapeutics, especially in high blood flow tumors, such as hepatocellular carcinoma. Currently, FDA-approved embolic microspheres include DC beads (Biocompatibles Inc, United Kingdom), HepaSpheres (Biosphere Medical Inc, America), CalliSpheres (Callisyn Biomedical Inc, China), etc. It is believed that more microspheres for different diseases will be approved for clinical studies in the future. Overall, the clinical translation process of microsphere delivery systems shows a trend of advancing year by year, but there are differences and multifaceted challenges in the direction of different disease applications.

As we continue to explore issues related to microsphere delivery strategies for the treatment of IVDD, we must be clear about one fact–the results achieved thus far are based on alternative models other than humans. Although rodent models of IVD, such as rats and rabbits, can partially recapitulate the pathological process of degeneration, their function and stress environment during daily activities are far different from human IVD (Liao et al., 2019). Therefore, in the search for a more realistic simulation, further experiments are necessary on upright walking animals such as monkeys and gorillas. In addition, the current research is biased toward exploring the efficacy of single factor improvement, which is not enough for IVDD with complex etiological mechanisms. Of course, much of this is due to limitations in available research techniques and inadequate understanding of the pathogenesis of IVDD. Furthermore, although NP tissue degeneration is a major component of IVDD, AF and CEP are equally not immune, and IVD should be improved as a whole motor unit. In summary, more reasonable animal models need to be selected when evaluating microsphere delivery systems, more adequately simulating the pathological state of the disease, and finally, implementing treatment for the overall structure. This will improve the safety, efficacy, and feasibility of IVD microsphere formulations and accelerate their transition to the clinic.

Moreover, the following issues must be considered for the clinical translation and even full-scale dissemination of microsphere therapy. First and foremost is the widespread concern about security. These microsphere delivery systems must undergo a rigorous safety evaluation to minimize human toxicity and other side effects as they move from the laboratory to the marketplace. Of these, the choice of materials is most critical. Some polymeric microspheres or composites doped with polymeric microspheres can trigger local tissue inflammatory responses when degraded *in vivo* (Shishatskaya et al., 2008; Habraken et al., 2010). This inflammatory effect and other side effects may be attributable to the tissue condition of the graft site and the composition and purity of the material, and careful selection of material is required if inflammation can, in turn, alter the rate of degradation and release of the microspheres. Second, the effectiveness of the product is crucial. However, most hydrophilic materials containing hydroxyl and carboxyl groups have low loading rates for hydrophobic substances (Liu et al., 2017). This greatly limits the drug loading efficiency of the microspheres. Additionally, it is largely difficult to replicate and predict the effects of complex and subtle environmental changes (e.g., oxygen levels, temperature, pH, and enzymes) in human tissues on microspheres during the testing of newly developed microsphere formulations (Lengyel et al., 2019). This may lead to less-than-expected sustained-release effects of the microspheres in human practice. Third, a certain stability is required to ensure the smooth functioning of the microspheres. Although microsphere-based injectable delivery systems excel in the sustained release of substances, there is a potential for migration from the injection site due to the lack of cohesion between the microspheres (Wang et al., 2012). Insufficient stability of the delivery system may be the reason for microsphere runoff from the treatment site, and increasing the adhesion between the microspheres is a viable option but requires a more demanding production technique. Finally, the production capacity of the microspheres is the basis for the implementation of related treatments. As we all know, the preparation of microspheres involves a series of complex processes and has strict requirements for equipment and production conditions and thus large-scale controlled production of microspheres is still challenging considering the cost of materials and time. Therefore, some commercially available microsphere products may be expensive. All of the above problems need to be addressed and solved so that microsphere therapy can be brought to more fields and better realize its clinical application.

## 5 Summary and Prospects

After reviewing the limitations of conventional therapies, we provide a comprehensive overview of the application of novel microsphere delivery systems in IVDD. Clearly, the excellent slow-release properties conferred by the porous structure, high encapsulation rate, and biodegradability, coupled with good surface adhesion, have led to the gradual emergence of microspheres as the preferred carrier in the field of tissue engineering. Such microsphere carriers allow targeted delivery and controlled release of drugs, gene regulatory sequences, and active substances, including proteins, suitable for localized diseases such as IVDD. Although still in the preclinical stage, microsphere-based delivery systems have shown outstanding therapeutic potential in IVDD. For example, microsphere delivery systems can largely avoid the end result of the explosive release and subsequent rapid clearance of therapeutic components in IVD, which is a strong guarantee for the smooth therapeutic effect of the loaded substances. In addition, microsphere-mediated sustained release effectively prolongs the local treatment time of substances, which is attractive for improving chronic disease. Furthermore, the delivery system does not stop alleviating the clinical symptoms of the disease, and its inhibition of disease progression and promotion of tissue regeneration are the ultimate therapeutic trends. In conclusion, the benefits of microsphere delivery systems include but are not limited to optimizing drug delivery strategies, extending treatment duration, inhibiting degenerative progression, and promoting tissue regeneration or repair.

Currently, there are few studies of microsphere therapies for IVDD, and many deficiencies remain to be further identified and refined. After ensuring the safety and efficacy of microsphere materials, the selection of therapeutic substances becomes crucial for effective microsphere therapy. The therapeutic components selected in all current microsphere delivery protocols support intervention in IVDD across multiple pathogenic mechanisms and achieve therapeutic objectives such as inhibition of inflammation, promotion of matrix synthesis, and restoration of ECM metabolic homeostasis. However, the singularity of interventions in single studies, the incomplete understanding of IVDD pathogenesis, and the lack of an effective targeted scheme have prevented the implementation of comprehensive treatment strategies with microspheres. In the future, a single study should block more possible etiologic mechanisms to expand the breadth and depth of microsphere therapy in IVDD. At the same time, more attention should be focused on the degenerating IVD tissue niche, as the inflammatory microenvironment of hypoxia, low nutrients, and low pH pose a huge challenge for microsphere delivery strategies. Moreover, the existing microsphere strategies focus on NP tissue while ignoring the treatment of AF and CEP, and such regenerative therapies that do not focus on the overall structure of the IVD may not yield substantial results, so future research should focus on the wholeness of IVD.

With the development and application of microsphere delivery systems in IVD tissue engineering, a large number of issues need to be properly addressed. This requires deeper learning and understanding of the pathological mechanisms of IVDD and the integration of IVD pathophysiology with IVD regenerative therapies to drive the clinical translation of microsphere delivery systems for use in IVDD. In the future, it may be possible to implement personalized treatment for patients at the mechanistic level using microsphere delivery technology through a well-established system for tracing the etiology of IVDD.

## Glossary

LBP: Low back pain
IVDD: Intervertebral disc degeneration
NP: Nucleus pulposus
AF: Annulus fibrosus
ECM: Extracellular matrix
CEP: Cartilaginous endplates
COL2: Type II collagen
PG: Proteoglycans
SASP: Senescence-associated secretory phenotype
MMP: Matrix metalloproteinase
TNF: Tumor necrosis factor
IL: Interleukin
ACAN: Aggrecan
NSAIDs: Nonsteroidal anti-inflammatory drugs
TDR: Total disc replacement
OP-1: Osteogenic protein 1
MSCs: Mesenchymal stem cells
3D: Three-dimensional
PLGA: Poly(lactic-co-glycolic acid)
PLLA: Poly(l-lactic acid)
PEA: Poly(ester amide)
W/O/W: Water-oil-water
GDF: Growth differentiation factor
TGF: Transforming growth factor
GelMA: Gelatin methacryloyl
ADSCs: Adipose-derived stem cells
ASCs: Adipose stem cells
GAG: Glycosaminoglycan
HA: Hyaluronic acid
rhsTNFRII: Recombinant human soluble tumor necrosis factor receptor II
DHI: Disc height index
ASIC-3: Acid-sensitive ion channel 3
PRP: Platelet rich plasma
DNPM: Decellularized nucleus pulposus matrix
CXB: Celecoxib
COX-2: Cyclooxygenase-2
PGE_2_: Prostaglandin E_2_
NGF: Nerve growth factor
TA: Triamcinolone acetonide
DEX: Dexamethasone
bFGF: Basic fibroblast growth factor
GlcNAc: N-acetyl-glucosamine
EGCG: Epigallocatechin 3-gallate
BMSCs: Bone marrow stromal cells
iPSCs: Induced pluripotent stem cells
NR4A1: Nuclear receptor 4A1

## References

[B1] Agnihotri N. Soni G. Chanchal D. K. Khan A. Tiwari S. (2019). A Review on Microspheres a Novel Drug Delivery System for Multiparticulate Drug Release. Int. J. Life Sci. Rev. 5, 6–15. 10.1016/j.jconrel.2018.08.019

[B2] Aihara T. Kojima A. Urushibara M. Endo K. Sawaji Y. Suzuki H. (2022). Long-term Reoperation Rates and Causes for Reoperations Following Lumbar Microendoscopic Discectomy and Decompression: 10-year Follow-Up. J. Clin. Neurosci. 95, 123–128. 10.1016/j.jocn.2021.11.015 34929635

[B3] Alkhatib B. Rosenzweig D. H. Rosenzweig D. Krock E. Roughley P. Beckman L. (2014). Acute Mechanical Injury of the Human Intervertebral Disc: Link to Degeneration and Pain. Eur. Cell Mater 28, 98–111. 10.22203/ecm.v028a08 25214017

[B4] Battié M. C. Haynor D. R. Fisher L. D. Gill K. Gibbons L. E. Videman T. (1995). Similarities in Degenerative Findings on Magnetic Resonance Images of the Lumbar Spines of Identical Twins. J. Bone & Jt. Surg. 77 (11), 1662–1670. 10.2106/00004623-199511000-00004 7593075

[B5] Battié M. C. Videman T. Levalahti E. Gill K. Kaprio J. (2007). Heritability of Low Back Pain and the Role of Disc Degeneration. Pain 131 (3), 272–280. 10.1016/j.pain.2007.01.010 17335977

[B6] Beatty S. (2018). We Need to Talk about Lumbar Total Disc Replacement. Int. J. Spine Surg. 12 (2), 201–240. 10.14444/5029 30276080 PMC6159637

[B7] Bian J. Cai F. Chen H. Tang Z. Xi K. Tang J. (2021). Modulation of Local Overactive Inflammation via Injectable Hydrogel Microspheres. Nano Lett. 21 (6), 2690–2698. 10.1021/acs.nanolett.0c04713 33543616

[B8] Bibby S. R. S. Jones D. A. Ripley R. M. Urban J. P. G. (2005). Metabolism of the Intervertebral Disc: Effects of Low Levels of Oxygen, Glucose, and pH on Rates of Energy Metabolism of Bovine Nucleus Pulposus Cells. Spine 30 (5), 487–496. 10.1097/01.brs.0000154619.38122.47 15738779

[B9] Birch J. Gil J. (2020). Senescence and the SASP: Many Therapeutic Avenues. Genes Dev. 34 (23-24), 1565–1576. 10.1101/gad.343129.120 33262144 PMC7706700

[B10] Boas F. E. Kemeny N. E. Sofocleous C. T. Yeh R. Thompson V. R. Hsu M. (2021). Bronchial or Pulmonary Artery Chemoembolization for Unresectable and Unablatable Lung Metastases: A Phase I Clinical Trial. Radiology 301 (2), 474–484. 10.1148/radiol.2021210213 34463550 PMC8574062

[B11] Bode B. W. McGill J. B. Lorber D. L. Gross J. L. Chang P.-C. Bregman D. B. (2015). Inhaled Technosphere Insulin Compared with Injected Prandial Insulin in Type 1 Diabetes: A Randomized 24-Week Trial. Diabetes Care 38 (12), 2266–2273. 10.2337/dc15-0075 26180109

[B12] Buser Z. Chung A. S. Abedi A. Wang J. C. (2019). The Future of Disc Surgery and Regeneration. Int. Orthop. (SICOT) 43 (4), 995–1002. 10.1007/s00264-018-4254-7 30506089

[B13] Cazzanelli P. Wuertz-Kozak K. (2020). MicroRNAs in Intervertebral Disc Degeneration, Apoptosis, Inflammation, and Mechanobiology. Int. J. Mol. Sci. 21 (10), 3601. 10.3390/ijms21103601 PMC7279351 32443722

[B14] Certo M. Tsai C.-H. Pucino V. Ho P.-C. Mauro C. (2021). Lactate Modulation of Immune Responses in Inflammatory versus Tumour Microenvironments. Nat. Rev. Immunol. 21 (3), 151–161. 10.1038/s41577-020-0406-2 32839570

[B15] Chang H. Cai F. Zhang Y. Jiang M. Yang X. Qi J. (2022). Silencing Gene‐Engineered Injectable Hydrogel Microsphere for Regulation of Extracellular Matrix Metabolism Balance. Small Methods 6, 2101201. 10.1002/smtd.202101201 34994105

[B16] Chang H. Wang H. Yang X. You K. Jiang M. Cai F. (2021). Comprehensive Profile Analysis of Differentially Expressed circRNAs in Glucose Deprivation-Induced Human Nucleus Pulposus Cell Degeneration. BioMed Res. Int. 2021, 4770792. 10.1155/2021/4770792 34285912 PMC8275381

[B17] Chang Y. Yang M. Ke S. Zhang Y. Xu G. Li Z. (2020). Effect of Platelet-Rich Plasma on Intervertebral Disc Degeneration *In Vivo* and *In Vitro*: A Critical Review. Oxidative Med. Cell. Longev. 2020, 8893819. 10.1155/2020/8893819 PMC7704139 33299533

[B18] Cheng Z. Xiang Q. Wang J. Zhang Y. (2021). The Potential Role of Melatonin in Retarding Intervertebral Disc Ageing and Degeneration: A Systematic Review. Ageing Res. Rev. 70, 101394. 10.1016/j.arr.2021.101394 34139338

[B19] Choi M. H. Blanco A. Stealey S. Duan X. Case N. Sell S. A. (2020). Micro-Clotting of Platelet-Rich Plasma upon Loading in Hydrogel Microspheres Leads to Prolonged Protein Release and Slower Microsphere Degradation. Polymers 12 (8), 1712. 10.3390/polym12081712 PMC7464943 32751604

[B20] Chow P. K. H. Gandhi M. Tan S.-B. Khin M. W. Khasbazar A. Ong J. (2018). SIRveNIB: Selective Internal Radiation Therapy versus Sorafenib in Asia-Pacific Patients with Hepatocellular Carcinoma. J. Clin. Oncol. 36 (19), 1913–1921. 10.1200/jco.2017.76.0892 29498924

[B21] Cieza A. Causey K. Kamenov K. Hanson S. W. Chatterji S. Vos T. (2021). Global Estimates of the Need for Rehabilitation Based on the Global Burden of Disease Study 2019: a Systematic Analysis for the Global Burden of Disease Study 2019. Lancet 396 (10267), 2006–2017. 10.1016/s0140-6736(20)32340-0 33275908 PMC7811204

[B22] Clarke L. E. McConnell J. C. Sherratt M. J. Derby B. Richardson S. M. Hoyland J. A. (2014). Growth Differentiation Factor 6 and Transforming Growth Factor-Beta Differentially Mediate Mesenchymal Stem Cell Differentiation, Composition, and Micromechanical Properties of Nucleus Pulposus Constructs. Arthritis Res. Ther. 16 (2), R67. 10.1186/ar4505 24618041 PMC4060243

[B23] Conaghan P. G. Cohen S. B. Berenbaum F. Lufkin J. Johnson J. R. Bodick N. (2018). Brief Report: A Phase IIb Trial of a Novel Extended‐Release Microsphere Formulation of Triamcinolone Acetonide for Intraarticular Injection in Knee Osteoarthritis. Arthritis Rheumatol. 70 (2), 204–211. 10.1002/art.40364 29088579 PMC5814922

[B24] Cui Y. Guo Y. Kong L. Shi J. Liu P. Li R. (2022). A Bone-Targeted Engineered Exosome Platform Delivering siRNA to Treat Osteoporosis. Bioact. Mater. 10, 207–221. 10.1016/j.bioactmat.2021.09.015 34901540 PMC8636739

[B25] Edeline J. Touchefeu Y. Guiu B. Farge O. Tougeron D. Baumgaertner I. (2020). Radioembolization Plus Chemotherapy for First-Line Treatment of Locally Advanced Intrahepatic Cholangiocarcinoma: A Phase 2 Clinical Trial. JAMA Oncol. 6 (1), 51–59. 10.1001/jamaoncol.2019.3702 31670746 PMC6824230

[B26] Eliasberg C. D. Kelly M. P. Ajiboye R. M. SooHoo N. F. (2016). Complications and Rates of Subsequent Lumbar Surgery Following Lumbar Total Disc Arthroplasty and Lumbar Fusion. Spine 41 (2), 173–181. 10.1097/brs.0000000000001180 26751061 PMC4710859

[B27] Feng C. Liu H. Yang M. Zhang Y. Huang B. Zhou Y. (2016). Disc Cell Senescence in Intervertebral Disc Degeneration: Causes and Molecular Pathways. Cell Cycle 15 (13), 1674–1684. 10.1080/15384101.2016.1152433 27192096 PMC4957599

[B28] Feng C. Liu H. Yang Y. Huang B. Zhou Y. (2015). Growth and Differentiation Factor-5 Contributes to the Structural and Functional Maintenance of the Intervertebral Disc. Cell Physiol. Biochem. 35 (1), 1–16. 10.1159/000369670 25547527

[B29] Feng G. Zhang Z. Dang M. Rambhia K. J. Ma P. X. (2020). Nanofibrous Spongy Microspheres to Deliver Rabbit Mesenchymal Stem Cells and Anti-miR-199a to Regenerate Nucleus Pulposus and Prevent Calcification. Biomaterials 256, 120213. 10.1016/j.biomaterials.2020.120213 32736170 PMC7423691

[B30] Feng G. Zhang Z. Dang M. Zhang X. Doleyres Y. Song Y. (2017). Injectable Nanofibrous Spongy Microspheres for NR4A1 Plasmid DNA Transfection to Reverse Fibrotic Degeneration and Support Disc Regeneration. Biomaterials 131, 86–97. 10.1016/j.biomaterials.2017.03.029 28376367 PMC5448136

[B31] Fontana G. Srivastava A. Thomas D. Lalor P. Dockery P. Pandit A. (2015). Three-Dimensional Microgel Platform for the Production of Cell Factories Tailored for the Nucleus Pulposus. Bioconjugate Chem. 26 (7), 1297–1306. 10.1021/bc5004247 25290910

[B32] Garin E. Tselikas L. Guiu B. Chalaye J. Edeline J. de Baere T. (2021). Personalised versus Standard Dosimetry Approach of Selective Internal Radiation Therapy in Patients with Locally Advanced Hepatocellular Carcinoma (DOSISPHERE-01): a Randomised, Multicentre, Open-Label Phase 2 Trial. Lancet Gastroenterology Hepatology 6 (1), 17–29. 10.1016/s2468-1253(20)30290-9 33166497

[B33] Ghosh Dastidar D. Saha S. Chowdhury M. (2018). Porous Microspheres: Synthesis, Characterisation and Applications in Pharmaceutical & Medical Fields. Int. J. Pharm. 548 (1), 34–48. 10.1016/j.ijpharm.2018.06.015 29940297

[B34] Gilbert H. T. J. Hodson N. Baird P. Richardson S. M. Hoyland J. A. (2016). Acidic pH Promotes Intervertebral Disc Degeneration: Acid-Sensing Ion Channel -3 as a Potential Therapeutic Target. Sci. Rep. 6, 37360. 10.1038/srep37360 27853274 PMC5112591

[B35] Gkantsinikoudis N. Kapetanakis S. Magras I. Tsiridis E. Kritis A. (Forthcoming 2022). Tissue Engineering of Human Intervertebral Disc: A Concise Review. Tissue Eng. Part B Rev. in Press. 10.1089/ten.TEB.2021.0090 34409867

[B36] Gorth D. J. Mauck R. L. Chiaro J. A. Mohanraj B. Hebela N. M. Dodge G. R. (2012). IL-1ra Delivered from Poly(lactic-Co-Glycolic Acid) Microspheres Attenuates IL-1β Mediated Degradation of Nucleus Pulposus *In Vitro* . Arthritis Res. Ther. 14 (4), R179. 10.1186/ar3932 22863285 PMC3580573

[B37] Gottlieb M. (2017). Statement by FDA Commissioner Scott Gottlieb, MD, on FDA Ushering in New Era of 3D Printing of Medical Products; Provides Guidance to Manufacturers of Medical Devices [WWW Document]. Available at: https://www.fda.gov/news-events/press-announcements/statement-fda-commissioner-scott-gottlieb-md-fda-ushering-new-era-3d-printing-medical-products (Accessed 3.15. 19).

[B38] Gu T. Shi Z. Wang C. Chen C. Wu J. Wang D. (2017). Human Bone Morphogenetic Protein 7 Transfected Nucleus Pulposus Cells Delay the Degeneration of Intervertebral Disc in Dogs. J. Orthop. Res. 35 (6), 1311–1322. 10.1002/jor.22995 26218641

[B39] Gu W. Zhu Q. Gao X. Brown M. D. (2014). Simulation of the Progression of Intervertebral Disc Degeneration Due to Decreased Nutritional Supply. Spine 39 (24), E1411–E1417. 10.1097/brs.0000000000000560 25188596 PMC4229452

[B40] Guerin H. L. Elliott D. M. (2007). Quantifying the Contributions of Structure to Annulus Fibrosus Mechanical Function Using a Nonlinear, Anisotropic, Hyperelastic Model. J. Orthop. Res. 25 (4), 508–516. 10.1002/jor.20324 17149747

[B41] Gupta V. Khan Y. Berkland C. J. Laurencin C. T. Detamore M. S. (2017). Microsphere-Based Scaffolds in Regenerative Engineering. Annu. Rev. Biomed. Eng. 19, 135–161. 10.1146/annurev-bioeng-071516-044712 28633566 PMC11610505

[B42] Habraken W. J. E. M. Liao H. B. Zhang Z. Wolke J. G. C. Grijpma D. W. Mikos A. G. (2010). *In Vivo* degradation of Calcium Phosphate Cement Incorporated into Biodegradable Microspheres. Acta Biomater. 6 (6), 2200–2211. 10.1016/j.actbio.2009.12.028 20026289

[B43] Harshitha S. M. Sibin M. K. Chetan G. K. Bhat D. I. (2018). Association of CILP, COL9A2 and MMP3 Gene Polymorphisms with Lumbar Disc Degeneration in an Indian Population. J. Mol. Neurosci. 66 (3), 378–382. 10.1007/s12031-018-1182-3 30288688

[B44] Hartvigsen J. Hancock M. J. Kongsted A. Louw Q. Ferreira M. L. Genevay S. (2018). What Low Back Pain Is and Why We Need to Pay Attention. Lancet 391 (10137), 2356–2367. 10.1016/s0140-6736(18)30480-x 29573870

[B45] Hassani A. Khoshfetrat A. B. Rahbarghazi R. Sakai S. (2022). Collagen and Nano-Hydroxyapatite Interactions in Alginate-Based Microcapsule Provide an Appropriate Osteogenic Microenvironment for Modular Bone Tissue Formation. Carbohydr. Polym. 277, 118807. 10.1016/j.carbpol.2021.118807 34893227

[B46] He Q. Zhang J. Liao Y. Alakpa E. V. Bunpetch V. Zhang J. (2020). Current Advances in Microsphere Based Cell Culture and Tissue Engineering. Biotechnol. Adv. 39, 107459. 10.1016/j.biotechadv.2019.107459 31682922

[B47] Henrotin Y. Marty M. Mobasheri A. (2014). What Is the Current Status of Chondroitin Sulfate and Glucosamine for the Treatment of Knee Osteoarthritis? Maturitas 78 (3), 184–187. 10.1016/j.maturitas.2014.04.015 24861964

[B48] Heo M. Park S. (2022). Biphasic Properties of PVAH (Polyvinyl Alcohol Hydrogel) Reflecting Biomechanical Behavior of the Nucleus Pulposus of the Human Intervertebral Disc. Materials 15 (3), 1125. 10.3390/ma15031125 35161069 PMC8838070

[B49] Hinkelmann S. Springwald A. H. Schulze S. Hempel U. Mitrach F. Wölk C. (2022). Mineralizing Gelatin Microparticles as Cell Carrier and Drug Delivery System for siRNA for Bone Tissue Engineering. Pharmaceutics 14 (3), 548. 10.3390/pharmaceutics14030548 35335924 PMC8949427

[B50] Hodgkinson T. Gilbert H. T. J. Pandya T. Diwan A. D. Hoyland J. A. Richardson S. M. (2020). Regenerative Response of Degenerate Human Nucleus Pulposus Cells to GDF6 Stimulation. Int. J. Mol. Sci. 21 (19), 7143. 10.3390/ijms21197143 PMC7582366 32992671

[B51] Hodgkinson T. Stening J. Z. White L. J. Shakesheff K. M. Hoyland J. A. Richardson S. M. (2019). Microparticles for Controlled Growth Differentiation Factor 6 Delivery to Direct Adipose Stem Cell‐based Nucleus Pulposus Regeneration. J. Tissue Eng. Regen. Med. 13 (8), 1406–1417. 10.1002/term.2882 31066515 PMC6771973

[B52] Hohaus C. Ganey T. M. Minkus Y. Meisel H. J. (2008). Cell Transplantation in Lumbar Spine Disc Degeneration Disease. Eur. Spine J. 17 (Suppl. 4), 492–503. 10.1007/s00586-008-0750-6 19005697 PMC2587656

[B53] Hoy D. March L. Brooks P. Blyth F. Woolf A. Bain C. (2014). The Global Burden of Low Back Pain: Estimates from the Global Burden of Disease 2010 Study. Ann. Rheum. Dis. 73 (6), 968–974. 10.1136/annrheumdis-2013-204428 24665116

[B54] Kaiser J. (2020). How Safe Is a Popular Gene Therapy Vector? Science 367 (6474), 131. 10.1126/science.367.6474.131 31919200

[B55] Kanayama M. Oha F. Hashimoto T. (2015). What Types of Degenerative Lumbar Pathologies Respond to Nerve Root Injection? A Retrospective Review of Six Hundred and Forty One Cases. Int. Orthop. (SICOT) 39 (7), 1379–1382. 10.1007/s00264-015-2761-3 25877160

[B56] Kang L. Xiang Q. Zhan S. Song Y. Wang K. Zhao K. (2019). Restoration of Autophagic Flux Rescues Oxidative Damage and Mitochondrial Dysfunction to Protect against Intervertebral Disc Degeneration. Oxidative Med. Cell. Longev. 2019, 27. 10.1155/2019/7810320 PMC6954474 31976028

[B57] Keshari K. R. Lotz J. C. Link T. M. Hu S. Majumdar S. Kurhanewicz J. (2008). Lactic Acid and Proteoglycans as Metabolic Markers for Discogenic Back Pain. Spine 33 (3), 312–317. 10.1097/BRS.0b013e31816201c3 18303465

[B58] Kong M. Zhang Y. Song M. Cong W. Gao C. Zhang J. (2021). Myocardin-related Transcription Factor A Nuclear Translocation Contributes to Mechanical Overload-induced Nucleus Pulposus Fibrosis in Rats with Intervertebral Disc Degeneration. Int. J. Mol. Med. 48 (1), 123. 10.3892/ijmm.2021.4956 33982787 PMC8121555

[B59] Kraus V. B. Conaghan P. G. Aazami H. A. Mehra P. Kivitz A. J. Lufkin J. (2018). Synovial and Systemic Pharmacokinetics (PK) of Triamcinolone Acetonide (TA) Following Intra-articular (IA) Injection of an Extended-Release Microsphere-Based Formulation (FX006) or Standard Crystalline Suspension in Patients with Knee Osteoarthritis (OA). Osteoarthr. Cartil. 26 (1), 34–42. 10.1016/j.joca.2017.10.003 29024802

[B60] Krupkova O. Ferguson S. J. Wuertz-Kozak K. (2016a). Stability of (−)-epigallocatechin Gallate and its Activity in Liquid Formulations and Delivery Systems. J. Nutr. Biochem. 37, 1–12. 10.1016/j.jnutbio.2016.01.002 27770867

[B61] Krupkova O. Handa J. Hlavna M. Klasen J. Ospelt C. Ferguson S. J. (2016b). The Natural Polyphenol Epigallocatechin Gallate Protects Intervertebral Disc Cells from Oxidative Stress. Oxidative Med. Cell. Longev. 2016, 17. 10.1155/2016/7031397 PMC4826942 27119009

[B62] Krupkova O. Sekiguchi M. Sekiguchi M. Klasen J. Hausmann O. Konno S. (2014). Epigallocatechin 3-gallate Suppresses Interleukin-1β-Induced Inflammatory Responses in Intervertebral Disc Cells *In Vitro* and Reduces Radiculopathic Pain in Rats. Eur. Cell Mater 28, 372–386. 10.22203/ecm.v028a26 25422948

[B63] Kulchar R. J. Denzer B. R. Chavre B. M. Takegami M. Patterson J. (2021). A Review of the Use of Microparticles for Cartilage Tissue Engineering. Int. J. Mol. Sci. 22 (19), 10292. 10.3390/ijms221910292 34638629 PMC8508725

[B64] Lengyel M. Kállai-Szabó N. Antal V. Laki A. J. Antal I. (2019). Microparticles, Microspheres, and Microcapsules for Advanced Drug Delivery. Sci. Pharm. 87 (3), 20. 10.3390/scipharm87030020

[B65] Li C. Bai Q. Lai Y. Tian J. Li J. Sun X. (2021). Advances and Prospects in Biomaterials for Intervertebral Disk Regeneration. Front. Bioeng. Biotechnol. 9, 766087. 10.3389/fbioe.2021.766087 34746112 PMC8569141

[B66] Li Y. Y. Diao H. J. Chik T. K. Chow C. T. An X. M. Leung V. (2014). Delivering Mesenchymal Stem Cells in Collagen Microsphere Carriers to Rabbit Degenerative Disc: Reduced Risk of Osteophyte Formation. Tissue Eng. Part A 20 (9-10), 1379–1391. 10.1089/ten.TEA.2013.0498 24372278 PMC4011461

[B67] Liang C.-z. Li H. Tao Y.-q. Peng L.-h. Gao J.-q. Wu J.-j. (2013). Dual Release of Dexamethasone and TGF-Β3 from Polymeric Microspheres for Stem Cell Matrix Accumulation in a Rat Disc Degeneration Model. Acta Biomater. 9 (12), 9423–9433. 10.1016/j.actbio.2013.08.019 23973308

[B68] Liang C. Z. Li H. Tao Y. Q. Zhou X. P. Yang Z. R. Xiao Y. X. (2012). Dual Delivery for Stem Cell Differentiation Using Dexamethasone and bFGF In/on Polymeric Microspheres as a Cell Carrier for Nucleus Pulposus Regeneration. J. Mater Sci. Mater Med. 23 (4), 1097–1107. 10.1007/s10856-012-4563-0 22327946

[B69] Liao Z. Luo R. Li G. Song Y. Zhan S. Zhao K. (2019). Exosomes from Mesenchymal Stem Cells Modulate Endoplasmic Reticulum Stress to Protect against Nucleus Pulposus Cell Death and Ameliorate Intervertebral Disc Degeneration *In Vivo* . Theranostics 9 (14), 4084–4100. 10.7150/thno.33638 31281533 PMC6592170

[B70] Lin J. Zhuge J. Zheng X. Wu Y. Zhang Z. Xu T. (2020). Urolithin A-Induced Mitophagy Suppresses Apoptosis and Attenuates Intervertebral Disc Degeneration via the AMPK Signaling Pathway. Free Radic. Biol. Med. 150, 109–119. 10.1016/j.freeradbiomed.2020.02.024 32105828

[B71] Lin T.-h. Tamaki Y. Pajarinen J. Waters H. A. Woo D. K. Yao Z. (2014). Chronic Inflammation in Biomaterial-Induced Periprosthetic Osteolysis: NF-Κb as a Therapeutic Target. Acta Biomater. 10 (1), 1–10. 10.1016/j.actbio.2013.09.034 24090989 PMC3849197

[B72] Liu L. Yao W. Rao Y. Lu X. Gao J. (2017). pH-Responsive Carriers for Oral Drug Delivery: Challenges and Opportunities of Current Platforms. Drug Deliv. 24 (1), 569–581. 10.1080/10717544.2017.1279238 28195032 PMC8241197

[B73] Loepfe M. Duss A. Zafeiropoulou K.-A. Björgvinsdóttir O. D’Este M. Eglin D. (2019). Electrospray-Based Microencapsulation of Epigallocatechin 3-Gallate for Local Delivery into the Intervertebral Disc. Pharmaceutics 11 (9), 435. 10.3390/pharmaceutics11090435 PMC6781552 31480533

[B74] Luan S. Wan Q. Luo H. Li X. Ke S. Lin C. (2015). Running Exercise Alleviates Pain and Promotes Cell Proliferation in a Rat Model of Intervertebral Disc Degeneration. Int. J. Mol. Sci. 16 (1), 2130–2144. 10.3390/ijms16012130 25607736 PMC4307353

[B75] Luan X. Tian X. Zhang H. Huang R. Li N. Chen P. (2019). Exercise as a Prescription for Patients with Various Diseases. J. Sport Health Sci. 8 (5), 422–441. 10.1016/j.jshs.2019.04.002 31534817 PMC6742679

[B76] Martin B. I. Mirza S. K. Spina N. Spiker W. R. Lawrence B. Brodke D. S. (2019). Trends in Lumbar Fusion Procedure Rates and Associated Hospital Costs for Degenerative Spinal Diseases in the United States, 2004 to 2015. Spine (Phila Pa 1976) 44 (5), 369–376. 10.1097/brs.0000000000002822 30074971

[B77] Masuda K. Imai Y. Okuma M. Muehleman C. Nakagawa K. Akeda K. (2006). Osteogenic Protein-1 Injection into a Degenerated Disc Induces the Restoration of Disc Height and Structural Changes in the Rabbit Anular Puncture Model. Spine 31 (7), 742–754. 10.1097/01.brs.0000206358.66412.7b 16582847

[B78] Mayer J. E. Iatridis J. C. Chan D. Qureshi S. A. Gottesman O. Hecht A. C. (2013). Genetic Polymorphisms Associated with Intervertebral Disc Degeneration. Spine J. 13 (3), 299–317. 10.1016/j.spinee.2013.01.041 23537453 PMC3655694

[B79] McCann M. R. Tamplin O. J. Rossant J. Séguin C. A. (2012). Tracing Notochord-Derived Cells Using a Noto-Cre Mouse: Implications for Intervertebral Disc Development. Dis. Model Mech. 5 (1), 73–82. 10.1242/dmm.008128 22028328 PMC3255545

[B80] McEvoy L. Carr D. F. Pirmohamed M. (2021). Pharmacogenomics of NSAID-Induced Upper Gastrointestinal Toxicity. Front. Pharmacol. 12, 684162. 10.3389/fphar.2021.684162 34234675 PMC8256335

[B81] McMillan A. Nguyen M. K. Gonzalez-Fernandez T. Ge P. Yu X. Murphy W. L. (2018). Dual Non-viral Gene Delivery from Microparticles within 3D High-Density Stem Cell Constructs for Enhanced Bone Tissue Engineering. Biomaterials 161, 240–255. 10.1016/j.biomaterials.2018.01.006 29421560 PMC5826638

[B82] Meisel H. J. Siodla V. Ganey T. Minkus Y. Hutton W. C. Alasevic O. J. (2007). Clinical Experience in Cell-Based Therapeutics: Disc Chondrocyte Transplantation A Treatment for Degenerated or Damaged Intervertebral Disc. Biomol. Eng. 24 (1), 5–21. 10.1016/j.bioeng.2006.07.002 16963315

[B83] Milette P. C. Fontaine S. Lepanto L. Cardinal É. Breton G. (1999). Differentiating Lumbar Disc Protrusions, Disc Bulges, and Discs with Normal Contour but Abnormal Signal Intensity. Magnetic Resonance Imaging with Discographic Correlations. Spine (Phila Pa 1976) 24 (1), 44–53. 10.1097/00007632-199901010-00011 9921590

[B84] Molladavoodi S. McMorran J. Gregory D. (2020). Mechanobiology of Annulus Fibrosus and Nucleus Pulposus Cells in Intervertebral Discs. Cell Tissue Res. 379 (3), 429–444. 10.1007/s00441-019-03136-1 31844969

[B85] Morris H. Gonçalves C. F. Dudek M. Hoyland J. Meng Q.-J. (2021). Tissue Physiology Revolving Around the Clock: Circadian Rhythms as Exemplified by the Intervertebral Disc. Ann. Rheum. Dis. 80 (7), 828–839. 10.1136/annrheumdis-2020-219515 33397731

[B86] Moser M. Adl Amini D. Jones C. Zhu J. Okano I. Oezel L. (2022). The Predictive Value of Psoas and Paraspinal Muscle Parameters Measured on MRI for Severe Cage Subsidence after Standalone Lateral Lumbar Interbody Fusion. Spine J. S1529-9430(22)00138-3. 10.1016/j.spinee.2022.03.009 35351664

[B87] Murab S. Samal J. Shrivastava A. Ray A. R. Pandit A. Ghosh S. (2015). Glucosamine Loaded Injectable Silk-In-Silk Integrated System Modulate Mechanical Properties in Bovine *Ex-Vivo* Degenerated Intervertebral Disc Model. Biomaterials 55, 64–83. 10.1016/j.biomaterials.2015.03.032 25934453

[B88] Naci H. Smalley K. R. Kesselheim A. S. (2017). Characteristics of Preapproval and Postapproval Studies for Drugs Granted Accelerated Approval by the US Food and Drug Administration. Jama 318 (7), 626–636. 10.1001/jama.2017.9415 28810023 PMC5817559

[B89] NaPier Z. Kanim L. E. A. Arabi Y. Salehi K. Sears B. Perry M. (2019). Omega-3 Fatty Acid Supplementation Reduces Intervertebral Disc Degeneration. Med. Sci. Monit. 25, 9531–9537. 10.12659/msm.918649 31836696 PMC6929565

[B90] Naqvi S. Gansau J. Gansau J. Gibbons D. Buckley C. (2019). *In Vitro* co-culture and *Ex Vivo* Organ Culture Assessment of Primed and Cryopreserved Stromal Cell Microcapsules for Intervertebral Disc Regeneration. Eur. Cell Mater 37, 134–152. 10.22203/eCM.v037a09 30768674

[B91] Naqvi S. M. Gansau J. Buckley C. T. (2018). Priming and Cryopreservation of Microencapsulated Marrow Stromal Cells as a Strategy for Intervertebral Disc Regeneration. Biomed. Mat. 13 (3), 034106. 10.1088/1748-605X/aaab7f 29380742

[B92] Paesold G. Nerlich A. G. Boos N. (2007). Biological Treatment Strategies for Disc Degeneration: Potentials and Shortcomings. Eur. Spine J. 16 (4), 447–468. 10.1007/s00586-006-0220-y 16983559 PMC2229827

[B93] Palumbo-Zerr K. Zerr P. Distler A. Fliehr J. Mancuso R. Huang J. (2015). Orphan Nuclear Receptor NR4A1 Regulates Transforming Growth Factor-β Signaling and Fibrosis. Nat. Med. 21 (2), 150–158. 10.1038/nm.3777 25581517

[B94] Park H.-J. Lee C.-S. Chung S.-S. Park S.-J. Kim W.-S. Park J.-S. (2018). Radiological and Clinical Long-Term Results of Heterotopic Ossification Following Lumbar Total Disc Replacement. Spine J. 18 (5), 762–768. 10.1016/j.spinee.2017.09.003 28939171

[B95] Patil P. Dong Q. Wang D. Chang J. Wiley C. Demaria M. (2019). Systemic Clearance of p16(INK4a)‐positive Senescent Cells Mitigates Age‐associated Intervertebral Disc Degeneration. Aging Cell 18 (3), e12927. 10.1111/acel.12927 30900385 PMC6516165

[B96] Peng Y. Qing X. Lin H. Huang D. Li J. Tian S. (2021). Decellularized Disc Hydrogels for hBMSCs Tissue-specific Differentiation and Tissue Regeneration. Bioact. Mater. 6 (10), 3541–3556. 10.1016/j.bioactmat.2021.03.014 33842740 PMC8022111

[B97] Ranjbar E. Namazi H. Pooresmaeil M. (2022). Carboxymethyl Starch Encapsulated 5-FU and DOX Co-loaded Layered Double Hydroxide for Evaluation of its *In Vitro* Performance as a Drug Delivery Agent. Int. J. Biol. Macromol. 201, 193–202. 10.1016/j.ijbiomac.2021.12.181 35007629

[B98] Ricke J. Klümpen H. J. Amthauer H. Bargellini I. Bartenstein P. de Toni E. N. (2019). Impact of Combined Selective Internal Radiation Therapy and Sorafenib on Survival in Advanced Hepatocellular Carcinoma. J. Hepatology 71 (6), 1164–1174. 10.1016/j.jhep.2019.08.006 31421157

[B99] Rosenstock J. Franco D. Korpachev V. Shumel B. Ma Y. Baughman R. (2015). Inhaled Technosphere Insulin versus Inhaled Technosphere Placebo in Insulin-Naïve Subjects with Type 2 Diabetes Inadequately Controlled on Oral Antidiabetes Agents. Diabetes Care 38 (12), 2274–2281. 10.2337/dc15-0629 26253730

[B100] Rudnik-Jansen I. Tellegen A. Beukers M. Öner F. Woike N. Mihov G. (2019). Safety of Intradiscal Delivery of Triamcinolone Acetonide by a Poly(esteramide) Microsphere Platform in a Large Animal Model of Intervertebral Disc Degeneration. Spine J. 19 (5), 905–919. 10.1016/j.spinee.2018.10.014 31056104

[B101] Sawamura K. Ikeda T. Nagae M. Okamoto S.-i. Mikami Y. Hase H. (2009). Characterization ofIn VivoEffects of Platelet-Rich Plasma and Biodegradable Gelatin Hydrogel Microspheres on Degenerated Intervertebral Discs. Tissue Eng. Part A 15 (12), 3719–3727. 10.1089/ten.TEA.2008.0697 19514846

[B102] Sayed N. Allawadhi P. Khurana A. Singh V. Navik U. Pasumarthi S. K. (2022). Gene Therapy: Comprehensive Overview and Therapeutic Applications. Life Sci. 294, 120375. 10.1016/j.lfs.2022.120375 35123997

[B103] Seaquist E. R. Blonde L. McGill J. B. Heller S. R. Kendall D. M. Bumpass J. B. (2020). Hypoglycaemia Is Reduced with Use of Inhaled Technosphere (®) Insulin Relative to Insulin Aspart in Type 1 Diabetes Mellitus. Diabet. Med. 37 (5), 752–759. 10.1111/dme.14202 31811662 PMC7216876

[B104] Shen J. Chen A. Cai Z. Chen Z. Cao R. Liu Z. (2022). Exhausted Local Lactate Accumulation via Injectable Nanozyme-Functionalized Hydrogel Microsphere for Inflammation Relief and Tissue Regeneration. Bioact. Mater. 12, 153–168. 10.1016/j.bioactmat.2021.10.013 35310385 PMC8897073

[B105] Shi J. Zhou X. Wang Z. Kurra S. Niu J. Yang H. (2019). Increased Lactic Acid Content Associated with Extracellular Matrix Depletion in a Porcine Disc Degeneration Induced by Superficial Annular Lesion. BMC Musculoskelet. Disord. 20 (1), 551. 10.1186/s12891-019-2937-x 31747924 PMC6868808

[B106] Shi S. Song S. Liu X. Zhao G. Ding F. Zhao W. (2022). Construction and Performance of Exendin-4-Loaded Chitosan-PLGA Microspheres for Enhancing Implant Osseointegration in Type 2 Diabetic Rats. Drug Deliv. 29 (1), 548–560. 10.1080/10717544.2022.2036873 35156499 PMC8856071

[B107] Shishatskaya E. I. Voinova O. N. Goreva A. V. Mogilnaya O. A. Volova T. G. (2008). Biocompatibility of Polyhydroxybutyrate Microspheres: *In Vitro* and *In Vivo* Evaluation. J. Mater Sci. Mater Med. 19 (6), 2493–2502. 10.1007/s10856-007-3345-6 18253816

[B108] Standaert C. J. Friedly J. Erwin M. W. Lee M. J. Rechtine G. Henrikson N. B. (2011). Comparative Effectiveness of Exercise, Acupuncture, and Spinal Manipulation for Low Back Pain. Spine (Phila Pa 1976) 36 (21 Suppl. l), S120–S130. 10.1097/BRS.0b013e31822ef878 21952184

[B109] Steele J. Bruce-Low S. Smith D. Osborne N. Thorkeldsen A. (2015). Can Specific Loading through Exercise Impart Healing or Regeneration of the Intervertebral Disc? Spine J. 15 (10), 2117–2121. 10.1016/j.spinee.2014.08.446 26409630

[B110] Sulaiman S. Chowdhury S. R. Fauzi M. B. Rani R. A. Yahaya N. H. M. Tabata Y. (2020). 3D Culture of MSCs on a Gelatin Microsphere in a Dynamic Culture System Enhances Chondrogenesis. Int. J. Mol. Sci. 21 (8), 2688. 10.3390/ijms21082688 PMC7215541 32294921

[B111] Sun K. Guo J. Yao X. Guo Z. Guo F. (2021). Growth Differentiation Factor 5 in Cartilage and Osteoarthritis: A Possible Therapeutic Candidate. Cell Prolif. 54 (3), e12998. 10.1111/cpr.12998 33522652 PMC7941218

[B112] Sun Z. Liu B. Luo Z.-J. (2020). The Immune Privilege of the Intervertebral Disc: Implications for Intervertebral Disc Degeneration Treatment. Int. J. Med. Sci. 17 (5), 685–692. 10.7150/ijms.42238 32210719 PMC7085207

[B113] Tellegen A. R. Rudnik-Jansen I. Beukers M. Miranda-Bedate A. Bach F. C. de Jong W. (2018). Intradiscal Delivery of Celecoxib-Loaded Microspheres Restores Intervertebral Disc Integrity in a Preclinical Canine Model. J. Control. Release 286, 439–450. 10.1016/j.jconrel.2018.08.019 30110616

[B114] Than K. D. Rahman S. U. Wang L. Khan A. Kyere K. A. Than T. T. (2014). Intradiscal Injection of Simvastatin Results in Radiologic, Histologic, and Genetic Evidence of Disc Regeneration in a Rat Model of Degenerative Disc Disease. Spine J. 14 (6), 1017–1028. 10.1016/j.spinee.2013.11.034 24291703 PMC4032598

[B115] Trefilova V. V. Shnayder N. A. Petrova M. M. Kaskaeva D. S. Tutynina O. V. Petrov K. V. (2021). The Role of Polymorphisms in Collagen-Encoding Genes in Intervertebral Disc Degeneration. Biomolecules 11 (9), 1279. 10.3390/biom11091279 34572492 PMC8465916

[B116] Tryfonidou M. A. de Vries G. Hennink W. E. Creemers L. B. (2020). "Old Drugs, New Tricks" - Local Controlled Drug Release Systems for Treatment of Degenerative Joint Disease. Adv. Drug Deliv. Rev. 160, 170–185. 10.1016/j.addr.2020.10.012 33122086

[B117] Tsaryk R. Gloria A. Russo T. Anspach L. De Santis R. Ghanaati S. (2015). Collagen-low Molecular Weight Hyaluronic Acid Semi-interpenetrating Network Loaded with Gelatin Microspheres for Cell and Growth Factor Delivery for Nucleus Pulposus Regeneration. Acta Biomater. 20, 10–21. 10.1016/j.actbio.2015.03.041 25861947

[B118] Urbańska J. Karewicz A. Nowakowska M. (2014). Polymeric Delivery Systems for Dexamethasone. Life Sci. 96 (1-2), 1–6. 10.1016/j.lfs.2013.12.020 24373835

[B119] Vadalà G. Sowa G. Hubert M. Gilbertson L. G. Denaro V. Kang J. D. (2012). Mesenchymal Stem Cells Injection in Degenerated Intervertebral Disc: Cell Leakage May Induce Osteophyte Formation. J. Tissue Eng. Regen. Med. 6 (5), 348–355. 10.1002/term.433 21671407

[B120] van Dijk B. G. M. Potier E. van Dijk M. Creemers L. B. Ito K. (2017). Osteogenic Protein 1 Does Not Stimulate a Regenerative Effect in Cultured Human Degenerated Nucleus Pulposus Tissue. J. Tissue Eng. Regen. Med. 11 (7), 2127–2135. 10.1002/term.2111 26612824

[B121] Vilgrain V. Pereira H. Assenat E. Guiu B. Ilonca A. D. Pageaux G. P. (2017). Efficacy and Safety of Selective Internal Radiotherapy with Yttrium-90 Resin Microspheres Compared with Sorafenib in Locally Advanced and Inoperable Hepatocellular Carcinoma (SARAH): an Open-Label Randomised Controlled Phase 3 Trial. Lancet Oncol. 18 (12), 1624–1636. 10.1016/s1470-2045(17)30683-6 29107679

[B122] Virmani T. Gupta J. (2017). Pharmaceutical Application of Microspheres: an Approach for the Treatment of Various Diseases. Int. J. Pharm. Sci. Res. 8 (8), 3253–3260. 10.13040/IJPSR.0975-8232.8(8).3252-60

[B123] Wang C. Zhang Y. Dong Y. (2021). Lipid Nanoparticle-mRNA Formulations for Therapeutic Applications. Acc. Chem. Res. 54 (23), 4283–4293. 10.1021/acs.accounts.1c00550 34793124 PMC10068911

[B124] Wang F. Cai F. Shi R. Wang X.-H. Wu X.-T. (2016). Aging and Age Related Stresses: a Senescence Mechanism of Intervertebral Disc Degeneration. Osteoarthr. Cartil. 24 (3), 398–408. 10.1016/j.joca.2015.09.019 26455958

[B125] Wang H. Leeuwenburgh S. C. G. Li Y. Jansen J. A. (2012). The Use of Micro- and Nanospheres as Functional Components for Bone Tissue Regeneration. Tissue Eng. Part B Rev. 18 (1), 24–39. 10.1089/ten.TEB.2011.0184 21806489 PMC3262980

[B126] Werner J. H. Rosenberg J. H. Keeley K. L. Agrawal D. K. (2018). Immunobiology of Periprosthetic Inflammation and Pain Following Ultra-high-molecular-weight-polyethylene Wear Debris in the Lumbar Spine. Expert Rev. Clin. Immunol. 14 (8), 695–706. 10.1080/1744666x.2018.1511428 30099915 PMC6287907

[B127] Wiersema T. Tellegen A. Beukers M. van Stralen M. Wouters E. van de Vooren M. (2021). Prospective Evaluation of Local Sustained Release of Celecoxib in Dogs with Low Back Pain. Pharmaceutics 13 (8), 1178. 10.3390/pharmaceutics13081178 34452138 PMC8398998

[B128] Wolff J. A. Malone R. W. Williams P. Chong W. Acsadi G. Jani A. (1990). Direct Gene Transfer into Mouse Muscle *In Vivo* . Science 247 (4949 Pt 1), 1465–1468. 10.1126/science.1690918 1690918

[B129] Wu P. H. Kim H. S. Jang I.-T. (2020). Intervertebral Disc Diseases PART 2: A Review of the Current Diagnostic and Treatment Strategies for Intervertebral Disc Disease. Int. J. Mol. Sci. 21 (6), 2135. 10.3390/ijms21062135 PMC7139690 32244936

[B130] Wu Y. Loaiza J. Banerji R. Blouin O. Morgan E. (2021). Structure‐function Relationships of the Human Vertebral Endplate. JOR Spine 4 (3), e1170. 10.1002/jsp2.1170 34611592 PMC8479528

[B131] Xia K. Zhu J. Hua J. Gong Z. Yu C. Zhou X. (2019). Intradiscal Injection of Induced Pluripotent Stem Cell-Derived Nucleus Pulposus-like Cell-Seeded Polymeric Microspheres Promotes Rat Disc Regeneration. Stem Cells Int. 2019, 1–14. 10.1155/2019/6806540 PMC6525958 31191679

[B132] Xu G. Liu C. Jiang J. Liang T. Yu C. Qin Z. (2020). A Novel Mechanism of Intervertebral Disc Degeneration: Imbalance between Autophagy and Apoptosis. Epigenomics 12 (13), 1095–1108. 10.2217/epi-2020-0079 32285684

[B133] Xu H. Sun M. Wang C. Xia K. Xiao S. Wang Y. (2020). Growth Differentiation Factor-5-Gelatin Methacryloyl Injectable Microspheres Laden with Adipose-Derived Stem Cells for Repair of Disc Degeneration. Biofabrication 13 (1), 015010. 10.1088/1758-5090/abc4d3 33361566

[B134] Xu J. Liu S. Wang S. Qiu P. Chen P. Lin X. (2019). Decellularised Nucleus Pulposus as a Potential Biologic Scaffold for Disc Tissue Engineering. Mater. Sci. Eng. C 99, 1213–1225. 10.1016/j.msec.2019.02.045 30889657

[B135] Xu S. Liu B. Fan J. Xue C. Lu Y. Li C. (2022). Engineered Mesenchymal Stem Cell-Derived Exosomes with High CXCR4 Levels for Targeted siRNA Gene Therapy against Cancer. Nanoscale 14 (11), 4098–4113. 10.1039/d1nr08170e 35133380

[B136] Xu W.-N. Zheng H.-L. Yang R.-Z. Liu T. Yu W. Zheng X.-F. (2019). Mitochondrial NDUFA4L2 Attenuates the Apoptosis of Nucleus Pulposus Cells Induced by Oxidative Stress via the Inhibition of Mitophagy. Exp. Mol. Med. 51 (11), 1–16. 10.1038/s12276-019-0331-2 PMC6861227 31740659

[B137] Xu Y. Gu Y. Cai F. Xi K. Xin T. Tang J. (2020). Metabolism Balance Regulation via Antagonist‐Functionalized Injectable Microsphere for Nucleus Pulposus Regeneration. Adv. Funct. Mat. 30 (52), 2006333. 10.1002/adfm.202006333

[B138] Yan J. Yang S. Sun H. Guo D. Wu B. Ji F. (2014). Effects of Releasing Recombinant Human Growth and Differentiation Factor-5 from Poly(lactic-Co-Glycolic Acid) Microspheres for Repair of the Rat Degenerated Intervertebral Disc. J. Biomater. Appl. 29 (1), 72–80. 10.1177/0885328213515034 24327349

[B139] Yang Y. Chen Q. Lin J. Cai Z. Liao G. Wang K. (2019). Recent Advance in Polymer Based Microspheric Systems for Controlled Protein and Peptide Delivery. Curr. Med. Chem. 26 (13), 2285–2296. 10.2174/0929867326666190409130207 30963961

[B140] Yeomans N. D. Graham D. Y. Husni M. E. Solomon D. H. Stevens T. Vargo J. (2018). Randomised Clinical Trial: Gastrointestinal Events in Arthritis Patients Treated with Celecoxib, Ibuprofen or Naproxen in the PRECISION Trial. Aliment. Pharmacol. Ther. 47 (11), 1453–1463. 10.1111/apt.14610 29667211

[B141] Yoo K. Thapa N. Chwae Y. Yoon S. Kim B. Lee J. (2022). Transforming Growth Factor-β Family and Stem Cell-Derived Exosome Therapeutic Treatment in Osteoarthritis (Review). Int. J. Mol. Med. 49 (5), 62. 10.3892/ijmm.2022.5118 35293597 PMC8930092

[B142] Yoshinaga N. Uchida S. Dirisala A. Naito M. Koji K. Osada K. (2022). Bridging mRNA and Polycation Using RNA Oligonucleotide Derivatives Improves the Robustness of Polyplex Micelles for Efficient mRNA Delivery. Adv. Healthc. Mater. 11 (9), 2102016. 10.1002/adhm.202102016 34913604

[B143] Yoshinaga N. Uchida S. Dirisala A. Naito M. Osada K. Cabral H. (2021). mRNA Loading into ATP-Responsive Polyplex Micelles with Optimal Density of Phenylboronate Ester Crosslinking to Balance Robustness in the Biological Milieu and Intracellular Translational Efficiency. J. Control. Release 330, 317–328. 10.1016/j.jconrel.2020.12.033 33359053

[B144] Yu Y. Wang Z. Wu S. Zhu C. Meng X. Li C. (2022). Glutathione-Sensitive Nanoglue Platform with Effective Nucleic Acids Gluing onto Liposomes for Photo-Gene Therapy. ACS Appl. Mat. Interfaces 14, 25126–25134. in press. 10.1021/acsami.2c04022 35608168

[B145] Yuan M. Yeung C. W. Li Y. Y. Diao H. Cheung K. M. C. Chan D. (2013). Effects of Nucleus Pulposus Cell-Derived Acellular Matrix on the Differentiation of Mesenchymal Stem Cells. Biomaterials 34 (16), 3948–3961. 10.1016/j.biomaterials.2013.02.004 23465833

[B146] Yurube T. Buchser W. J. Moon H. J. Hartman R. A. Takayama K. Kawakami Y. (2019). Serum and Nutrient Deprivation Increase Autophagic Flux in Intervertebral Disc Annulus Fibrosus Cells: an *In Vitro* Experimental Study. Eur. Spine J. 28 (5), 993–1004. 10.1007/s00586-019-05910-9 30847707 PMC6538458

[B147] Zehra U. Robson-Brown K. Adams M. A. Dolan P. (2015). Porosity and Thickness of the Vertebral Endplate Depend on Local Mechanical Loading. Spine 40 (15), 1173–1180. 10.1097/brs.0000000000000925 25893360

[B148] Zhan J.-W. Wang S.-Q. Feng M.-S. Gao J.-H. Wei X. Yu J. (2021). Effects of Axial Compression and Distraction on Vascular Bud and VEGFA Expression in the Vertebral Endplate of an *Ex Vivo* Rabbit Spinal Motion Segment Culture Model. Spine (Phila Pa 1976) 46 (7), 421–432. 10.1097/brs.0000000000003816 33186278

[B149] Zhan J.-W. Wang S.-Q. Feng M.-S. Wei X. Yu J. Yin X.-L. (2020). Constant Compression Decreases Vascular Bud and VEGFA Expression in a Rabbit Vertebral Endplate *Ex Vivo* Culture Model. PLoS One 15 (6), e0234747. 10.1371/journal.pone.0234747 32584845 PMC7316323

[B150] Zhang H.-Y. Zhang Y. Zhang Y. Jiang Z.-P. Cui Y.-L. Wang Q.-S. (2022). ROS-Responsive Thioketal-Linked Alginate/chitosan Carriers for Irritable Bowel Syndrome with Diarrhea Therapy. Int. J. Biol. Macromol. 209 (Pt A), 70–82. 10.1016/j.ijbiomac.2022.03.118 35351547

[B151] Zhang N.-Z. Xiong Q.-S. Yao J. Liu B.-L. Zhang M. Cheng C.-K. (2022). Biomechanical Changes at the Adjacent Segments Induced by a Lordotic Porous Interbody Fusion Cage. Comput. Biol. Med. 143, 105320. 10.1016/j.compbiomed.2022.105320 35183971

[B152] Zhang T.-W. Li Z.-F. Dong J. Jiang L.-B. (2020). The Circadian Rhythm in Intervertebral Disc Degeneration: an Autophagy Connection. Exp. Mol. Med. 52 (1), 31–40. 10.1038/s12276-019-0372-6 31983731 PMC7000407

[B153] Zhang W. Sun T. Li Y. Yang M. Zhao Y. Liu J. (2022). Application of Stem Cells in the Repair of Intervertebral Disc Degeneration. Stem Cell Res. Ther. 13 (1), 70. 10.1186/s13287-022-02745-y 35148808 PMC8832693

[B154] Zhang X.-B. Hu Y.-C. Cheng P. Zhou H.-Y. Chen X.-Y. Wu D. (2021). Targeted Therapy for Intervertebral Disc Degeneration: Inhibiting Apoptosis Is a Promising Treatment Strategy. Int. J. Med. Sci. 18 (13), 2799–2813. 10.7150/ijms.59171 34220308 PMC8241771

[B155] Zhang, Y. Y. Yang B. Wang J. Cheng F. Shi K. Ying L. (2020). Cell Senescence: A Nonnegligible Cell State under Survival Stress in Pathology of Intervertebral Disc Degeneration. Oxidative Med. Cell. Longev. 2020, 1–12. 10.1155/2020/9503562 PMC7479476 32934764

[B156] Zhao L. Manchikanti L. Kaye A. D. Abd-Elsayed A. (2019). Treatment of Discogenic Low Back Pain: Current Treatment Strategies and Future Options-A Literature Review. Curr. Pain Headache Rep. 23 (11), 86. 10.1007/s11916-019-0821-x 31707499

[B157] Zhou Z. Cui S. Du J. Richards R. G. Alini M. Grad S. (2021). One Strike Loading Organ Culture Model to Investigate the Post-traumatic Disc Degenerative Condition. J. Orthop. Transl. 26, 141–150. 10.1016/j.jot.2020.08.003 PMC7773974 33437633

[B158] Zhu K. Zhao F. Yang Y. Mu W. (2020). Effects of Simvastatin-loaded PLGA Microspheres on Treatment of Rats with Intervertebral Disk Degeneration and on 6-K-PGF1α and HIF-1α. Exp. Ther. Med. 19 (1), 579–584. 10.3892/etm.2019.8267 31897100 PMC6923742

